# Pterostilbene attenuates microglial inflammation and brain injury after intracerebral hemorrhage in an OPA1-dependent manner

**DOI:** 10.3389/fimmu.2023.1172334

**Published:** 2023-08-08

**Authors:** Yang Wu, Qing Hu, Xiaoliang Wang, Hongbo Cheng, Jiegang Yu, Yang Li, Jianing Luo, Qingjiu Zhang, Jianliang Wu, Gengshen Zhang

**Affiliations:** ^1^Department of Neurosurgery, The Second Hospital of Hebei Medical University, Shijiazhuang, Hebei, China; ^2^Department of Neurosurgery, Tangdu Hospital, Air Force Medical University, Xi’an, Shaanxi, China; ^3^Department of Neurosurgery, The General Hospital of Hebei Medical University, Shijiazhuang, Hebei, China; ^4^Department of Neurosurgery, West Theater General Hospital, Chengdu, Sichuan, China

**Keywords:** intracerebral hemorrhage, pterostilbene, neuroinflammation, microglia, mitochondria, OPA1

## Abstract

Microglial activation and subsequent inflammatory responses are critical processes in aggravating secondary brain injury after intracerebral hemorrhage (ICH). Pterostilbene (3’, 5’-dimethoxy-resveratrol) features antioxidant and anti-inflammation properties and has been proven neuroprotective. In this study, we aimed to explore whether Pterostilbene could attenuate neuroinflammation after experimental ICH, as well as underlying molecular mechanisms. Here, a collagenase-induced ICH in mice was followed by intraperitoneal injection of Pterostilbene (10 mg/kg) or vehicle once daily. PTE-treated mice performed significantly better than vehicle-treated controls in the neurological behavior test after ICH. Furthermore, our results showed that Pterostilbene reduced lesion volume and neural apoptosis, and alleviated blood-brain barrier (BBB) damage and brain edema. RNA sequencing and subsequent experiments showed that ICH-induced neuroinflammation and microglial proinflammatory activities were markedly suppressed by Pterostilbene treatment. With regard to the mechanisms, we identified that the anti-inflammatory effects of Pterostilbene relied on remodeling mitochondrial dynamics in microglia. Concretely, Pterostilbene reversed the downregulation of OPA1, promoted mitochondrial fusion, restored normal mitochondrial morphology, and reduced mitochondrial fragmentation and superoxide in microglia after OxyHb treatment. Moreover, conditionally deleting microglial OPA1 in mice largely countered the effects of Pterostilbene on alleviating microglial inflammation, BBB damage, brain edema and neurological impairment following ICH. In summary, we provided the first evidence that Pterostilbene is a promising agent for alleviating neuroinflammation and brain injury after ICH in mice, and uncovered a novel regulatory relationship between Pterostilbene and OPA1-mediated mitochondrial fusion.

## 1 Introduction

Intracerebral hemorrhage (ICH), as the most devastating and least treatable form of stroke, causes high mortality and disability worldwide (1, 2). There are two pathological processes following ICH: primary brain injury and secondary brain injury, which contribute to varying degrees and kinds of life-long disabilities in most survivors (3, 4). The current therapies focusing on the primary injury have limited success, which has led to a center of attention on secondary injury mechanisms (5, 6). Increasing evidence has shown that neuroinflammation is the critical process in the aggravation of secondary injury after ICH, which is the main reason for poor prognosis (4), and ongoing inflammatory responses can aggravate BBB damage, neuronal death and neurological impairment (7). Microglia, as resident immune surveillance cells of the central nervous system, assist in maintaining brain homeostasis (8). After ICH occurs, microglia are quickly activated and secrete numerous inflammatory cytokines into the extracellular setting to trigger an overwhelming inflammatory reaction and exacerbate secondary brain injury, eventually contributing to the worse prognosis of experimental animals after ICH (4). Therefore, this process may determine the destiny of neuroinflammation. However, the mechanisms underlying microglial activation are not fully clear, and effective intervention is needed to explore (9, 10).

Mitochondria represent vital organelles that receive various signals from cells to produce ATP and regulate energy homeostasis in response to environmental and physiological alterations (11). However, mounting evidence suggests that mitochondria also integrate and transmit information to initiate adaptive changes in cells (12). In numerous studies, mitochondrial damage emerged as the main cause of microglial activation and neuroinflammation in various neurological disorders, such as stroke, TBI, and Alzheimer’s disease (AD) (13–15). Under such stressful conditions, impaired mitochondria can release various inflammation-promoting signals, including reactive oxygen species (ROS), mitochondria-derived peptides, Ca2^+^, cytochrome c, and some yet-to-be-characterized signals, ultimately leading to the inflammatory response (16). Notably, the significance of mitochondrial dynamics in regulating mitochondrial function and cellular physiology gained attention in recent years, as an increasing number of studies were conducted. On top of that, research suggested that impairment of mitochondrial dynamics is also a crucial factor in microglial activation and inflammation (17, 18). For example, the disruption of mitochondrial dynamics leads to the generation of proinflammatory mediators such as NF-kB and mitogen-activated protein kinase (MAPK), causing microglia to polarize towards the M1 type (19). Drp1 and Mff are critical regulators of mitochondrial fission, while MFN1, MFN2, and OPA1 regulate mitochondrial fusion (20); all four molecules can be targeted to regulate inflammatory processes. As more data confirm that mitochondrial dynamics play significant roles in the regulation of microglial function, it provides a novel therapeutic approach for ischemic stroke, AD, neurodegenerative diseases, inflammatory and autoimmune diseases (21–24). However, the role of mitochondrial dynamics in microglial activation after ICH is still largely unknown (3, 25). These data inspired us to analyze the mechanisms of the microglia-derived inflammatory response after ICH from the perspective of mitochondrial dynamics, which may provide a novel therapeutic direction for ICH.

Pterostilbene (3′, 5′-dimethoxy-resveratrol, PTE), as a natural stilbene derived from resveratrol, shows higher oral bioactivity and bioavailability (26, 27). This polyphenol is safe for humans and exerts counteractive effects against ageing, cancer, diabetes, oxidation, depression, and nerve injury (26). Intriguingly, PTE can cross the BBB and has been proven to have neuroprotective effects in many neurological diseases (27, 28). Previous studies have discovered the benefits of PTE in inhibiting inflammatory and mitochondrial oxidative stress injury after cerebral ischemic stroke (27), as well as alleviating inflammation and oxidation-involved early brain injury following subarachnoid hemorrhage (SAH) (29). PTE has also been found to exert potent neuromodulatory effects on ageing and AD through the regulation of neurogenesis and neuroinflammation (30). Moreover, recent studies have revealed that PTE could suppress microglia-mediated inflammation in neurological disorders such as neurodegenerative diseases, trauma, and cerebral ischemia (26, 31, 32). Regarding the mechanisms, PTE has been discovered to suppress microglial activation through numerous pathways, including the inhibition of the NLRP3/caspase-1 inflammasome pathway, activation of the SIRT-1 signaling pathway, the NF-κB signaling pathway, and others (26, 33, 34). Nevertheless, the impact of PTE on microglial polarization and neuroinflammatory damage in ICH remains undisclosed. If proven effective, elucidating the underlying mechanisms is imperative for further exploration.

The main objective of the present study was to explore the potential therapeutic effects of PTE on secondary brain injury in the ICH model. We utilized a widely accepted collagenase-induced ICH mouse model, which involves injecting bacterial collagenase into the striatum to induce blood vessel rupture and bleeding, mimicking the physiological processes of spontaneous bleeding in human brain blood vessels and the pathological changes of hematoma expansion following bleeding that evolves over hours. And we confirmed the protective effect of the PTE treatment through comprehensive evaluations such as behavioral tests, magnetic resonance imaging, apoptosis, brain edema, and others. Furthermore, we also aimed to evaluate the roles of PTE in suppressing microglia-mediated inflammation. Additionally, the study set out to explore the effects of PTE on mitochondrial fission and fusion, mitochondrial biogenesis, ATP synthesis and oxidative stress in OxyHb-treated microglia, as well as underlying molecular mechanisms.

## 2 Materials and methods

### 2.1 Animals and ethics

Adult male wild-type C57BL/6J mice (8 weeks, 20-25 g) were offered by the Animal Center of Air Force Medical University, OPA1-floxp mice by Cyagen (Cyagen Biosciences), and CX3CR1-CreERT2 mice by Gempharmatech Co., Ltd. Then, Tamoxifen injection in OPA1^fl/fl^/Cx3Cr1-CreERT2 mice induced conditional knockout of OPA1 in microglia. All mice were adapted for 1 week in a temperature-constant room in 12-h light/12-h dark cycles. The mice were given free access to water and food. All experiments complied with the guidelines of the National Institutes of Health Guide for the Care and Use of Laboratory Animals. The study protocol was approved by the Ethics Committee of our institutional organs.

### 2.2 ICH surgery and mediation

An ICH mouse model was established through the induction of collagenase (3). The principle is as follows (35, 36): Collagenase is a metalloproteinase that can specifically degrade collagen in the extracellular matrix and basement membrane of blood vessels. It is distributed around cerebral blood vessels and can cause vascular damage when activated, leading to leakage of blood into surrounding tissues and the formation of cerebral hemorrhage. The mouse was placed in a stereoscopic brain locator (D01453-003, Rayward Life Science, Shenzhen, China) after being deeply anesthetized intraperitoneally using 2% pentobarbital sodium. ICH was induced by stereotaxic injection of collagenase into the right striatum. After disinfection and incision, the skull was drilled to create a 0.5-mm-diameter hole that allowed for the insertion of a needle into the corpus striatum. For the collagenase-induced model, a total of 0.2 µl of 0.075U type VII-S collagenase (C2399, Sigma-Aldrich) in saline was injected at a rate of 0.05 µl/min under stereotactic guidance (injection coordinates, 0.20 mm anterior; 2.30 mm right lateral; 3.20 mm deep). Then, the needle stayed for an additional 5 min to prevent reflux, and was slowly removed before the hole was sealed. The sham group received the same procedure except for the infusion with collagenase. The dead mice (~15–20% mortality) or ICH model mice without neurological deficits were excluded from the following analysis. Then, experimental mice were averaged into three groups: sham group, ICH group, and ICH + PTE group.

**Figure 1:**
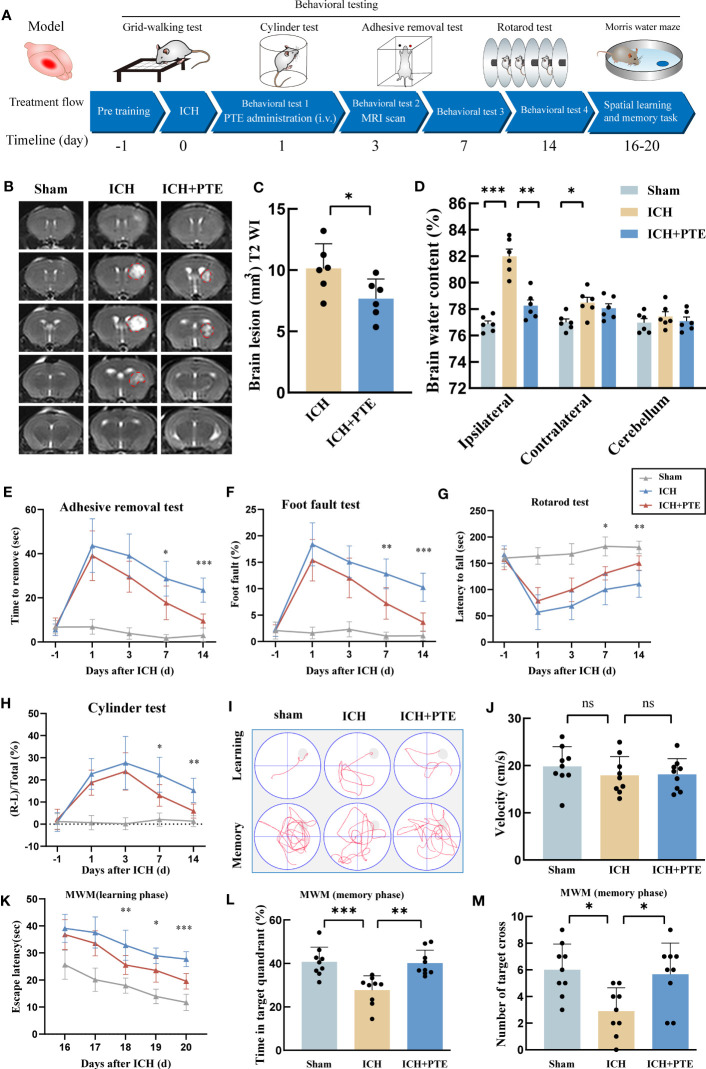
Effects of PTE on hemorrhage volume and neurological function after ICH. **(A)** Experimental schematic. **(B)** Representative axial T2 images gained from each group at 3 d after ICH. Red lines delineate lesion areas (n = 6 per group). **(C)** Quantification of hematoma volumes on T2-weighted images. **(D)** Quantification of brain water content at 3 d after ICH (n = 6 per group). **(E–H)** Sensorimotor functions were measured by adhesive removal **(E)**, foot fault **(F)**, rotarod **(G)** and cylinder test **(H)** at 1, 3, 7, and 14 d after ICH (ICH+PTE group vs ICH group, n = 9 per group). **(I)** The spatial memory and learning ability were measured by the Morris water maze test. **(J)** Graph showing the swim speed of mice in the different groups during the screening test before the water maze learning sessions (n = 9 per group). **(K)** Escape latency in the Morris water maze test (ICH+PTE group vs ICH group, n = 9 per group). **(L, M)** Time spent in the target quadrant and the number of platform crossings during the probe trial in which the target platform is removed (n = 9 per group). Values are expressed as mean ± SD. Significance was determined by Student’s t-test **(C)** and one-way **(J, L, M)** or two-way repeated ANOVA **(D, E–H, K)** with Bonferroni *post hoc* tests. *P < 0.05, **P < 0.01, **P < 0.001. In behavioral experiments, the asterisks on the top indicate single day comparison between the two groups (ICH+PTE group vs ICH group).

PTE (purity ≥98.0%; Sigma-Aldrich, St. Louis, MO, USA) was prepared in dimethyl sulfoxide (DMSO), followed by dilution with saline in advance. Following the different group allocations, the mice were treated with either vehicle or PTE (10 mg/kg, intraperitoneally) at 1 h after surgery, according to previous studies (27, 29). After receiving different treatments, the mice were sacrificed after 72 h for histological and molecular biologic assays or treated with vehicle or PTE daily for behavioral testing (**Figure 1**). Sham groups were administered with normal saline containing DMSO.

### 2.3 Measurement of lesion volume

Lesion volume in the brain was measured at post-ICH 3d by a 3T small animal MRI scanner. The brain was scanned and sliced into equally spaced coronal sections. The T2‐weighted image sequence was used to calculate lesion volume by multiplying the sum of the area by the distance between every two sections.

### 2.4 Brain water content

The brain tissues were dissected and divided into three parts: ipsilateral, contralateral hemispheres, and cerebellums, all weighed for their wet weight. Then, the tissues were dried at 95-100°C for 72 h to measure the dry weight. Tissue water content = (wet weight − dry weight)/wet weight × 100%.

### 2.5 Neurobehavioral tests

All neurobehavioral tests were performed by two experimenters who were blinded to the grouping of the animals. The recordings were made using a video camera placed above or under the test area, and the videos were later analyzed by a third experimenter who was also blinded to the grouping of the animals.

The foot fault test was used to assess the ability of mice to place their paws on the grid (6). The mouse was placed on a metal wire grid surface and videotaped for 1 min from beneath the grid. The videotape was used to analyze the number of total steps taken, and the number of foot faults made by the left limbs. Foot faults were referred to once the mouse misplaced its left forepaw or hind paw, such that the paw slipped off the grid. The percentage of foot faults in total steps made by that paw was calculated. The test was performed at 1 d before the surgery, and repeated at 1, 3, 7, and 14 d after surgery.

The adhesive removal test was used to assess forepaw sensitivity and motor impairments (6). Tapes (2×3-mm) were applied to the left forepaw. The tactile response was quantified as the time to remove the adhesive with its mouth during a maximum of 120 sec. The test was performed at 1 d before surgery, and repeated at 1, 3, 7, and 14 d after surgery.

The cylinder test was used to assess the asymmetry between the activities of forelimbs (6). The mouse was placed individually inside a transparent cylinder and videotaped from the top for 5 min. The times for both or each forelimb to support the body during rearing and lateral exploration were recorded by videotapes. The asymmetry was calculated as (right-left)/(left + right + both) × 100%. The test was performed at 1 d before surgery, and repeated at 1, 3, 7, and 14 d after surgery.

The rotarod test was used to assess sensorimotor coordination (6). The mouse was placed on a rod, with speed increasing linearly from 4 to 40 rpm. Each mouse underwent three trials with a 5-min intermission in between. The latency to fall was scored in seconds. The values from the three trials were averaged. The test was performed at 1 d before surgery, and repeated at 1, 3, 7, and 14 d after surgery.

Morris water maze test (MWM) was used to assess the spatial memory and learning ability of mice (37). Briefly, the mice (male, 2 months old) were trained twice daily with an interval of 1 h from 16 d to 21 d after ICH. During the learning phase in the first 5 days, the latency to find the hidden platform within 60 s was recorded. Then, the memory phase test was carried out at 21 d. In the probe trial test and navigation test, the mice were allowed to swim for 1 min after the removal of the platform. The time spent in the targeted quadrant and the number of platform crossovers were measured and compared.

### 2.6 BBB permeability

BBB permeability was assessed using Evans blue dye extravasation at 3 d after ICH (3). Having been injected with Evans blue (4ml/kg) in saline via the tail vein at 3 h before dissection, the mice were treated with a high dose of 2% pentobarbital sodium, then transcardially perfused with 0.9% cold saline. Brain tissues were weighed, sliced into 6 equally spaced coronal sections for photographs, homogenized with saline, and centrifugated (12,000×g, 30 min). Next, we transferred an equal volume of trichloroacetic acid to the resultant supernatant. After overnight incubation (4°C), the supernatant was centrifuged once again (12,000×g, 30 min). Lastly, the extravasation of Evans blue dye was quantified by spectrophotometric analysis (620 nm).

### 2.7 Tissue preparation

At post-ICH 72 h, the mice were anesthetized and sacrificed, as mentioned above (3). Then the mice received transcardiac perfusion of 4% paraformaldehyde. The brain tissues were removed and stored in 4% paraformaldehyde at 4°C overnight, then dehydrated with 10%, 20%, and 30% sucrose solutions, respectively. The brain tissues were sliced into 15-25 μm sections for further analyses and experiments. We selected brain sections at -0.3 to +0.5 mm anterior to bregma. Then, we identified tissue within 1 mm of the hematoma as perihematomal tissue used for immunofluorescence analysis and TUNEL staining.

### 2.8 Immunofluorescence and terminal deoxynucleotidyl transferase-mediated dUTP nick end labeling (TUNEL) staining

The samples, made as mentioned above, were incubated for 30 min in phosphate buffered saline (PBS) solution containing 0.1% Triton X-100 and blocked for 30 min in TBS containing 5% goat serum (Gibco, USA). Then, the sections were incubated at 4°C overnight with primary antibodies IBA1 (1:500; Abcam) and CD68 (1:200; Abcam). Next, the sections were soaked in PBS and incubated with corresponding secondary antibodies for 1 h at 25°C. Lastly, the sections were dyed for 15 min in DAPI (1:1000, Invitrogen) to label nuclei at 25°C. An A1 Si confocal microscope (Nikon, Japan) was used to image all sections.

Tunel staining was used to detect DNA fragmentation in the perihematomal brain tissues using the *In Situ* Cell Death Detection Kit (Roche, Germany). TUNEL-positive cells were counted. The ratio of TUNEL-positive to DAPI-stained cells was calculated to evaluate the levels of apoptosis.

### 2.9 Microglia isolation by flow cytometry

The brains were dissected, and the perihematomal tissue was collected in cold phosphate-buffered saline (PBS). Subsequently, the tissues were dissociated at 37°C for a duration of 1 hour using Papain (2 mg ml-1, Worthington) in RPMI 1640 medium (Gibco). Following this, the dispersed cells were filtered through a 70 mm nylon mesh and meticulously collected through centrifugation. The cells were then resuspended in a 30% Percoll density gradient (GE Healthcare) and subjected to centrifugation for 25 minutes at 900 g and 25°C to effectively isolate the cells in the lower fraction of the 30% Percoll solution. Prior to the staining procedure, the samples were blocked utilizing FcR Blocking Reagent (Miltenyi Biotec). The cells were subsequently washed and resuspended in PBS containing 2% fetal bovine serum (FBS). The evaluation of surface antigens in mouse microglia was carried out using flow cytometry, as previously documented (38–40). FITC anti-mouse CD11b Antibody (eBioscience) and APC-eFluor™ 780 anti-mouse CD45 Antibody (eBioscience) were utilized to stain the cells. Fluorescence acquisition was subsequently performed employing the FACS Aria II SORP instrument (BD Biosciences, USA), and the acquired data were analyzed using FlowJo_V10 software. For each antibody, gating was determined based on the appropriate negative isotype stained controls.

### 2.10 RNA-seq and downstream analyses

At post-ICH 72 h, mice were anesthetized, and brain tissue was dissected as described previously. Brain tissue surrounding the visible hematoma was sliced into 1mm sections using a blade on ice. Tissue located within 1-2 mm of the hematoma was identified as perihematomal tissue and used for RNA-seq analysis. Corresponding brain tissue from the Sham group mice, which were not subjected to injury, was collected as a control group. After cooling by placing surrounding tissue into liquid nitrogen, we transfered the tissue to a -80°C freezer for sequencing. The mRNA-seq method was briefly described as follows: RNA was extracted using Trizol reagent (thermofisher, 15596018). Library preparation of samples was performed using the Illumina TruSeq Stranded mRNA Sample Preparation Kit (Illumina Novaseq 6000 [LC-Bio Technology CO., Ltd., Hangzhou, China]). Fastq files for each tissue were mapped against the mouse genome (Mus_musculus.GRCm38) using the HISAT2 (https://daehwankimlab.github.io/hisat2/, version:hisat2-2.2.1) package with default parameter settings. rRNA transcripts were removed from the mapped bam files. Expression levels for each tissue were quantified using StringTie (http://ccb.jhu.edu/software/stringtie/, version:stringtie-2.1.6), which estimates the raw counts and FPKM values for each gene. FPKM values were used for the correlation plot to assess the global expression profile. The raw counts from the cuffdiff were subsequently used to quantify the differential expression levels for genes using the package DESeq2. Multiple testing correction was done using using the free online platform of Lianchuan Cloud Platform (https://www.omicstudio.cn/). LogFC of the genes was plotted against their log10 (qvalues) as a volcano plot to give an overview of significant DE genes. Hierarchical clustering of different tissues was performed using log2 transformed CPM values and heatmaps plotted using the above Cloud Platform.

### 2.11 Quantitative Real-Time PCR (qRT-PCR)

According to the manufacturer’s protocol, total RNA was extracted with TRIzol Reagent (Invitrogen, USA), and then was reverse-transcribed with HiScript II Q RT SuperMix for qRT-PCR (Vazyme, China) (3). An iQTM 5 Optical Module Real-Time PCR Detection System (Bio-Rad, USA) was used to perform qRT-PCR. According to the manufacturer’s instructions, the ChamQTM SYBR qPCR master mix (Vazyme biotech, China) was used to quantify mRNA. The primers used are listed in **Table S1**. The mRNA levels were normalized to β-actin and calculated using 2-ΔΔCt method.

### 2.12 Cell culture and treatment

The BV2 cells were cultured (37°C, 5% CO2) in Dulbecco’s modified Eagle’s high-glucose medium with 10% fetal bovine serum, 100 U/ml penicillin, and 100 µg/ml streptomycin in cell culture incubator. The vehicle group was exposed to an equivalent volume of PBS, and the OxyHb group to 30 μM oxyhemoglobin (OxyHb, Solarbio) for 24 h to mimic ICH (41, 42). OxyHb + PTE group was treated with PTE (5 µM) for 4 h before OxyHb treatment (26). Cell transfection with siRNA targeting mouse OPA1 (si-OPA1) and control scrambled siRNA (si-NC) was conducted using the Lipo2000 Reagent (Invitrogen, Carlsbad, CA, USA) according to the manufacturer’s instructions (43). The gene primers are shown in **Table S1**.

### 2.13 Analysis of mitochondrial morphology and function

BV2 cells on a confocal petri dish were subjected to the appropriate treatments. Thereafter, the cells were incubated for 30 minutes in serum-free cell culture medium with 10 nM MitoTracker Red (Life Technologies, USA) (44). Mitochondrial morphology was analyzed using a fluorescent microscope. Then, the mitochondrial function was analyzed by measuring mitochondrial ROS, ATP production and mitochondrial membrane potential (MMP). The cells were incubated with 5 nM MitoSOX (Invitrogen) or 10 nM JC-1 (Beyotime) for 30 minutes (3, 45). The sections were captured by a fluorescent microscope, and the relative fluorescence levels were quantified. ATP content (BC0300, Solarbio) was assayed using the appropriate commercially available kits, following the manufacturer’s instructions (45).

### 2.14 Enzyme-Linked Immunosorbent Assay (ELISA) for IL-1β, IL-18, IL-6, and TNF-α Detection in Brains and Cell Culture Supernatants

After BV2 microglia being treated with different conditions, culture supernatants were collected for ELISA analysis. Then, the culture supernatant was centrifuged at 15,000 × g for 5 min to remove the particulate matter, and directly used in fresh tubes. The concentrations of IL-1β, IL-18, IL-6, and TNF-α were detected by commercially available enzyme-linked immunosorbent assay kits (Biolegend, United States) according to the manufacturer’s protocol.

### 2.15 Western blot analysis

After euthanasia as described above, the circulating blood cells were removed from brain tissue through transcardial perfusion with PBS. Samples of the mouse tissue and BV2 cells were sonicated and homogenized by an ultrasonic homogenizer in the lysis buffer comprising 1% protease and phosphatase inhibitor (Roche, Mannheim, Germany). After lysate centrifugation, the supernatant was collected with its concentration measured by the BCA Protein Assay Kit (Thermo Scientific; UA276918). Using sodium dodecyl sulfate-polyacrylamide gel electrophoresis (SDSPAGE) (30 μg of protein per well of the electrophoresis gel), the protein was isolated, then transferred to polyvinylidene fluoride (PVDF) membranes (Millipore, Billerica, MA, USA), followed by treatment with 5% non-fat milk in Tris-buffered saline and Tween 20 (TBST) (pH 7.6). Then, the primary antibodies were used to incubate membranes overnight at 4°C, followed by incubation with the horseradish peroxidase-conjugated secondary antibodies at room temperature for 2 h. Lastly, the membranes were washed with TBST three times, 5 min each time. The protein bands were detected using a Bio-Rad imaging system (Bio-Rad, Hercules, CA, USA). The following primary antibodies were used: anti -Claudin5(1:800, ab131259, Abcam); anti-Occludin (1:1000, ab216327, Abcam); anti-Bax(1:1000, 50599, Proteintech); anti-Bcl2(1:1000, 12789, Proteintech); anti-β-actin(1:3000, AC026, ABclonal); anti-OPA1(1/1000, ab157457, Abcam); anti-MFN1(1:1000, ab221661, Abcam); anti-MFN2(1:2000, ab124773, Abcam); anti-Drip(1:1000, D6C7, Cell Signaling)

### 2.16 Statistical analysis

We presented the data by mean ± standard deviation (SD) unless otherwise noted, and the Shapiro-Wilk normality test was used to assess the normality of the data. If the data showed homogeneity and normality of variance, Student’s t-test for two-group differences or one-way ANOVA for multiple-group comparison with Bonferroni *post hoc* test was performed. The t-test or one-way ANOVA with Welch’s correction was used for normally distributed data with unequal variances. If the data were not in a normal distribution, the Mann-Whitney U test for two-group differences and non-parametric statistics (Kruskal-Wallis H) for multiple groups were performed. Besides, two-way repeated ANOVA was used to analyze the data of neurological function. Statistical significance was considered as *P* < 0.05. GraphPad Prism 9.0 software (GraphPad, USA) was used to analyze the data.

## 3 Results

### 3.1 The effects of PTE on lesion volume and neurological function scores after ICH

To determine the neuroprotective effects of PTE on ICH-induced brain injury, we first measured lesion volume by MRI and conducted behavioral testing in different groups respectively. MRI results showed that the lesion volume in ICH+PTE group significantly decreased as compared to that of ICH group at post-ICH 3 d (**Figures 1B, C**; ICH vs ICH+PTE, t =2.339, *P* = 0.0414). Besides, PTE treatment significantly enhanced sensorimotor functions. In the adhesive removal test, PTE treatment reduced the time spent removing the adhesive tape from the impaired forepaw at 1, 3, 7 and 14 d after ICH surgery, as compared to ICH group (**Figure 1E**; ICH vs ICH+PTE, F=116.7, p<0.0001). Comparable findings were observed in the grid-walking test that PTE reduced foot faults at 1, 3, 7 and 14 d after ICH, as compared to ICH group (**Figure 1F**; ICH vs ICH+PTE, F=80.53, p<0.0001). This therapeutic effect was also proven in the latency to fall and cylinder test (**Figures 1G, H**; Latency to fall test: ICH vs ICH+PTE, F=58.26, P<0.0001; Cylinder test: ICH vs ICH+PTE, F=43.65, P<0.0001). Furthermore, spatial learning and memory were assessed by the MWM at 16, 17, 18, 19, 20, and 21 d after ICH (**Figure 1I**). There was no significant difference between these groups in swimming speed (**Figure 1J**; F=0.6612, P=0.5254), while the escape latency increased in ICH group versus sham, which was reversed by PTE (**Figure 1K**; ICH vs ICH+PTE, F=159.6, P<0.0001). Meanwhile, PTE improved spatial memory, as indicated by the increased time in the target quadrant (**Figure 1L**; ICH vs ICH+PTE, F=11.83, p=0.0012) and numbers of platform crossing (**Figure 1M**; ICH vs ICH+PTE, F= 6.373, p=0.006) during the probe phase of MWM after PTE treatment. Consistent with the MWM behavioral test results, we found that neuronal loss was observed in the hippocampus after ICH injury by Nissl staining (CA1, F=27.66, P<0.0001; CA2, F=45.50, P<0.0001; CA3, F=12.89, P=0.0005), while PTE treatment significantly rescued the loss of neurons compared to the ICH group (CA1, F=27.66, P=0.0013; CA2, F=45.50, P<0.0001; CA3, F=12.89, P=0.0135) (**Supplementary Figure 1**). These results showed that PTE exerted neuroprotective effects against ICH insults in mice.

### 3.2 The effects of PTE on BBB damage, brain water content and neural apoptosis after ICH

**Figure 2:**
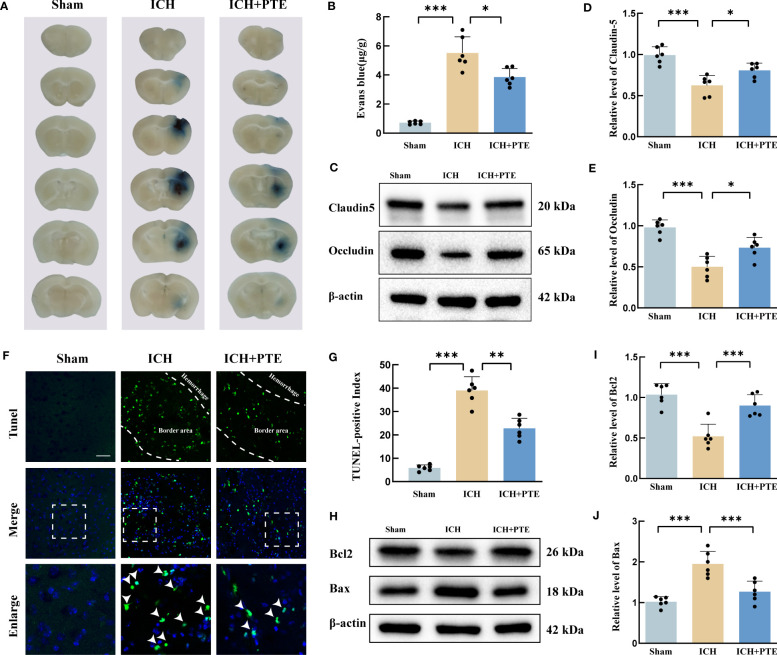
PTE ameliorated BBB impairment, brain edema and neural apoptosis after ICH. **(A)** Representative brain sections with Evan blue showing different extravasation of dye in each group at 3 d after ICH. **(B)** Quantification of Evan Blue in each group. **(C–E)** Western blot assay to detect the expression of Claudin-5 and Occludin in each group, and the related statistical analysis. **(F)** Representative TUNEL staining images, Scale bar, 50 μm. **(G)** Cell death was quantified by the TUNEL-positive index calculated by dividing the number of TUNEL-positive cells (green) by the total cell number (blue) at 3 d after ICH. **(H, I, J)** Representative Western blot images of Bax and Bcl-2. The densities of the protein bands were analyzed and normalized to β-actin. Values are mean ± SD (n = 6 per group). Significance was determined by one-way ANOVA **(B, D, E, G, I, J)** with Bonferroni *post hoc* tests. *P < 0.05, **P < 0.01, ***P < 0.001.

Since BBB impairment is a critical process in ICH-induced secondary brain injury, we investigated whether PTE could protect against BBB damage. The results showed a noticeable elevation in Evans blue extravasation in the ICH group, but PTE significantly inhibited this elevation (**Figures 2A, B**; ICH vs Sham, F=120.3, *P*=0.0004; ICH vs ICH+PTE, F=120.3, *P*=0.0337), indicating that PTE could protect BBB integrity against ICH injury. We further found that the expression of tight junction proteins (Claudin-5 and Occludin) in ICH group was lower than that in sham group, which was also reversed by PTE (**Figures 2C–E**; Claudin-5: ICH vs. Sham, F=120.3, *P*<0.0001; ICH vs ICH+PTE, F=120.3, *P*=0.0260; Occludin: ICH vs Sham, F=25.36, *P*<0.0001; ICH vs ICH+PTE, F=25.36, *P*=0.0102). Meanwhile, brain edema was assessed by the percentage of brain water content. We found that ICH-induced brain edema decreased significantly in PTE-treated groups (**Figure 1D**; ipsilateral: ICH vs Sham, F=28.72, P=0.0002; ICH vs ICH+PTE, F=28.72, P=0.0011; Contralateral: ICH vs Sham, F=28.72, P=0.0368).

Then, TUNEL staining revealed that ICH significantly increased the ratio of positive cells, which was repressed by PTE (**Figures 2F, G**; ICH vs Sham, F=116.5, *P*=0.0001; ICH vs ICH+PTE, F=116.5, *P* =0.0009). Meanwhile, Western blotting displayed an evident rise in Bax and a fall in Bcl2 induced by ICH, both significantly reversed by PTE (**Figures 2H–J**; Bcl2: ICH vs Sham, F=21.81, *P*<0.0001; ICH vs ICH+PTE, F=116.5, *P* =0.0009; Bax: ICH vs Sham, F=23.60, *P*<0.0001; ICH vs ICH+PTE, F=23.60, *P* =0.0006). Briefly, our data indicated that after ICH, PTE treatment exerted neuroprotective effects against BBB damage and anti-apoptotic effects.

### 3.3 PTE inhibited microglial activation and suppressed neuroinflammation after ICH

**Figure 3:**
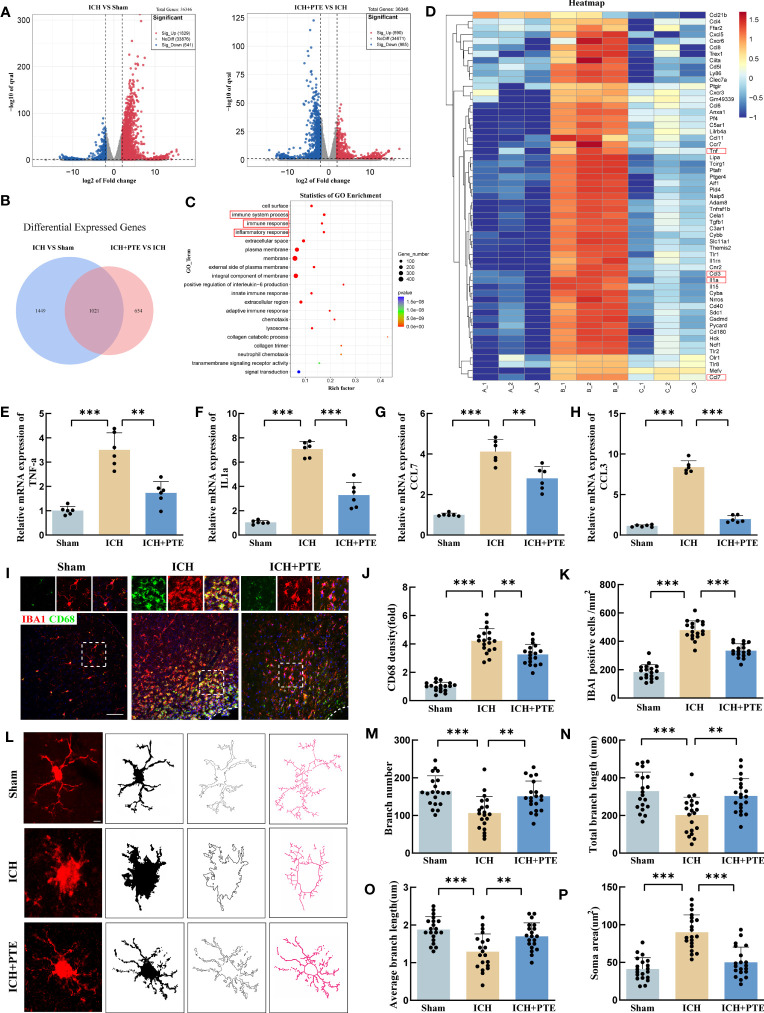
PTE suppressed microglial proinflammatory activities and attenuated neuroinflammation injury after ICH. **(A)** Different gene distribution was clearly seen on the volcano plot. **(B)** Venn diagram showed PTE reversed the expression of 1021 genes. **(C)** GO enrichment analysis of the overlap of genes in the Venn diagram. **(D)** Heat map showed the changes in the expression of inflammation-related genes in each group. **(E–H)** qPCR analysis of TNF-α, IL-1α, CCL7 and CCL3 (n = 6 per group). **(I)** Representative immunofluorescence staining of Iba1-positive cells within the peripheral tissue in each group. Scale bar, 100 μm. **(J)** Quantification analysis of the relative fluorescence intensity in microglia (18 fields of view). **(K)** The number of microglia was counted (18 fields of view). **(L)** Skeleton analysis of microglia morphologies in Iba1 stained tissue: Statistical analysis of **(M)** branch numbers, **(N)** total branch length (micrometers), **(O)** average branch length (micrometers), and **(P)** soma area (square micrometers) of Iba1-positive cells in each group, Scale bar, 10 μm, (n = 20 from 6 mice per group). Values are mean ± SD. Significance was determined by one-way ANOVA **(F, G, I–L, N–P)** with Bonferroni *post hoc* tests. *P < 0.05, **P < 0.01, ***P < 0.001.

To investigate the impact of PTE on ICH injury at the molecular level, we employed RNA sequencing (RNA-seq) to obtain a differential transcriptome profile of the mRNA expression under different treatments. The volcano plots clearly displayed the distribution of differentially expressed genes (DEGs) (**Figure 3A**). Venn diagram showed that compared to sham group, ICH group presented 2470 DEGs, and the 1021 genes of them were reversed by PTE treatment (**Figure 3B**). As depicted in **Figure 3C**, GO analysis highlighted inflammation-related processes as the most enriched pathways, including immune system process, immune response, inflammatory response. Then, a heat map showed that most DEGs were involved in these pathways, suggesting that PTE exerted its protective effect through regulating inflammatory response (**Figure 3D**). Many inflammation pathway-related genes were altered after PTE treatment. The four representative inflammatory factors (TNF-α, IL1α, CCL7, CCL3) were then selected for further study. In the qPCR results (**Figures 3E–H**), PTE significantly inhibited the activation of those inflammatory factors induced by ICH (TNF-α: ICH vs Sham, F=36.84, *P*=0.0004; ICH vs ICH+PTE, F=36.84, *P* =0.0018; IL1α: ICH vs Sham, F=256.4, *P*<0.0001; ICH vs. ICH+PTE, F=256.4, *P* =0.0002; CCL7: ICH vs Sham, F=62.82, *P*=0.0001; ICH vs ICH+PTE, F=62.82, *P* =0.0088; CCL3: ICH vs. Sham, F=343.8, P<0.0001; ICH vs ICH+PTE, F=62.82, P<0.0001). Immunofluorescence analysis showed the level of CD68, a marker of microglial hyperactivation, significantly elevated with the microglia showing an augmented cell size and increasing number after ICH, all indicators significantly reversed by PTE (**Figures 3I–K**; CD68 density: ICH vs Sham, F=160, P<0.0001; ICH vs ICH+PTE, F=160, P=0.0030; IBA1 positive cells: ICH vs Sham, F=125.2, P<0.0001; ICH vs ICH+PTE, F=125.2, P<0.0001). To investigate the effects of PTE on additional markers of activated microglia (**Supplementary Figure 2**), specifically the M1 type (CD86, INOS) and M2 type (CD206, Arg1), microglia were sorted using flow cytometry, and the expression of these markers in microglia was assessed through qPCR analysis. Our results showed that PTE treatment significantly decreased the transcription levels of CD86 and INOS (INOS, F=120.3, P<0.0001; CD86, F=83.92, P<0.0001; CD206, F=76.32, P<0.0001; Arg1, F=179.1, P<0.0001), while simultaneously increasing the transcription levels of CD206 and Arg1 in mice following ICH (INOS, F=120.3, P<0.0001; CD86, F=83.92, P<0.0001; CD206, F=76.32, P<0.0001; Arg1, F=179.1, P<0.0001). These findings suggest that PTE effectively inhibits microglial polarization towards proinflammatory phenotype. After Iba1 staining, the microglial morphology was delineated and skeletonized using the ImageJ software (**Figure 3L**). ICH increased the soma area, and decreased the branch number, average length and total length of microglia branches, which were significantly rescued by PTE treatment (**Figures 3M–P**; Branch number: ICH vs. Sham, F=10.72, P=0.0001; ICH vs ICH+PTE, F=10.72, P=0.0042; Total length of microglia branches: ICH vs Sham, F=9.672, P=0.0003; ICH vs ICH+PTE, F=9.672, P=0.0046; Average length of microglia branches: ICH vs Sham, F=11.7, P<0.0001; ICH vs ICH+PTE, F=11.7, P=0.0055; Soma area: ICH vs Sham, F=34.81, P<0.0001; ICH vs ICH+PTE, F=34.81, P<0.0001). In all, PTE inhibited microglial activation and suppressed neuroinflammation after ICH.

### 3.4 PTE promoted OPA1-mediated mitochondrial fusion in microglia after ICH

**Figure 4:**
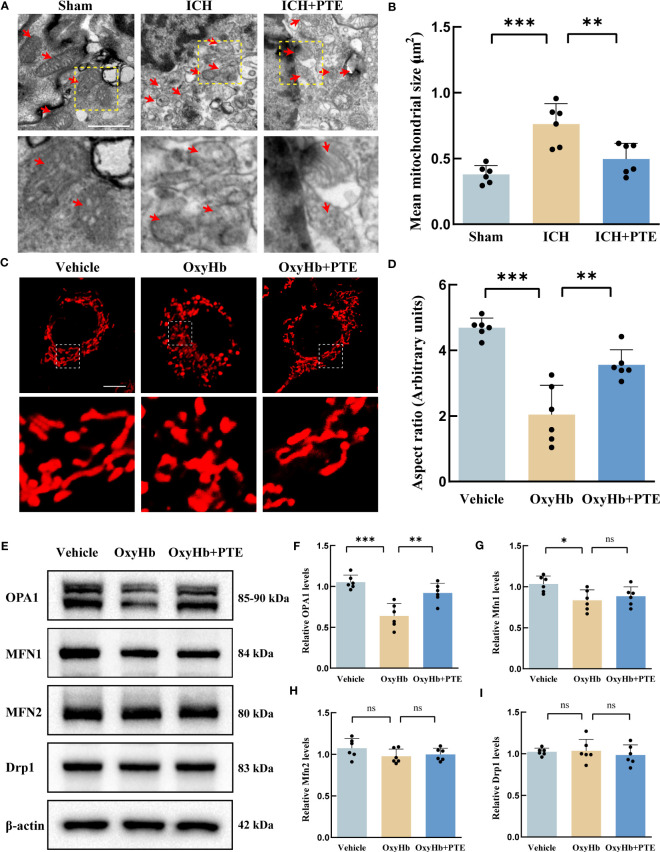
PTE promoted OPA1-mediated mitochondrial fusion in microglia after ICH. **(A)** mitochondrial ultrastructure in microglia revealed by *in vivo* electron microscopy. The boxed area next to each micrograph shows an amplified version of the white square. Scale bar = 1 μm. **(B)** Quantitative data for mitochondrial size. **(C)** To simulate ICH in the brain, we exposed BV2 microglia to 30 μM OxyHb for 24 hours. Changes in mitochondrial morphology in BV2 microglia through MitoTracker staining in each group. Each micrograph next to the boxed area shows an amplified version of the white square. Scale bar = 10 μm. **(D)** Mitochondrial fragmentation was quantified as aspect ratio (the ratio of mitochondrial length to width in BV2 microglia) by ImageJ software. **(E–I)** Western blot assay to detect the expression of OPA1, MFN1, MFN2, and DRP1 in BV2 microglia with different treatments and the related statistical analysis. Values are expressed as mean ± SD (n = 6 per group). Significance was determined by one-way ANOVA **(B, D, F–I)** with Bonferroni *post hoc* tests. *P < 0.05, **P < 0.01, ***P < 0.001, ns, no significance.

To explore the anti-inflammatory mechanism of PTE, we first investigated ultrastructural changes in microglia by transmission electron micrographs *in vivo*. The sham group showed some globular mitochondrial structures and long tubular mitochondrial structures accompanied by prominent cristae in microglia. However, the mitochondrial morphology was seriously impaired after ICH, featuring the loss of mitochondrial cristae, collapsed cristae and small mitochondrial size, which were markedly reversed by additional PTE treatment (**Figures 4A, B**; ICH vs. Sham, F=16.13, P=0.0002; ICH vs. ICH+PTE, F=16.13, P=0.0049). To further explore its underlying mechanism, we performed *in vitro* experiments. We exposed BV2 microglia to 30 μM OxyHb for 24 hours to construct an *in vitro* model. Firstly, we validated the protective effect of PTE on mitochondrial morphology in the *in vitro* model using MitoTracker staining, as shown in **Figures 4C, D**. MitoTracker staining results showed that ICH group significantly decreased the aspect ratio, an index of mitochondrial fragmentation, which was restored by 5 µM PTE treatment (**Figures 4C, D**; Vehicle vs OxyHb, F=29.16, P<0.0001; OxyHb vs OxyHb+PTE, F=29.16, P=0.0017). As Mfn1, Mfn2, OPA1 and Drp1 are primary manipulators of mitochondrial dynamics, we measured the protein expression of OPA1, Mfn1, Mfn2 and Drp1 in microglia. We observed that PTE significantly enhanced the protein expression of OPA1 after OxyHb treatment (**Figures 4E, F**; Vehicle vs OxyHb, F=18.45, P<0.0001; OxyHb vs OxyHb+PTE, F=18.45, P=0.0017), but had no influence on that of Mfn1, Mfn2, and Drp1 (**Figures 4G–I**; Mfn1: Vehicle vs OxyHb, F=5.063, P=0.238; OxyHb vs OxyHb+PTE, F=5.063, P>0.9999; Mfn2: Vehicle vs OxyHb, F=1.693, P=0.2985; OxyHb vs OxyHb+PTE, F=1.693, P>0.9999; Drp1: Vehicle vs OxyHb, F=0.3569, P>0.9999; OxyHb vs OxyHb+PTE, F=0.3569, P>0.9999). Meanwhile, we found that OxyHb treatment resulted in a decrease in OPA1 mRNA levels (F=6.680, P=0.0105), while PTE administration led to an increase in OPA1 mRNA levels (F=6.680, P>0.0443) (**Supplementary Figure 3A**). This suggests that PTE regulates OPA1 expression at the transcriptional level. Nrf2 is a central regulator of mitochondrial morphology, function, and mitophagy (46–48), and recent studies have indicated that Opa1 is a target gene of Nrf2 (49, 50). We further conducted a thorough investigation into the expression levels of Nrf2 in BV2 cells, specifically in the cytoplasm and nucleus (shown in **Supplementary Figure 3B**). Our findings revealed a significant decrease in Nrf2 levels in BV2 cells following OxyHb treatment, while the administration of PTE resulted in enhanced Nrf2 levels, both in the cytoplasm and nucleus (**Figure 3B**; cytosolic Nrf2: Vehicle vs OxyHb, F=9.526, P=0.0023; OxyHb vs OxyHb+PTE, F=9.526, P=0.0203; nuclear Nrf2: Vehicle vs OxyHb, F=30.83, P<0.0001; OxyHb vs OxyHb+PTE, F=30.83, P=0.0082), indicating a promoting effect of PTE on Nrf2 expression. Moreover, we knocked down Nrf2 in BV2 microglia and analyzed OPA1 expression in different groups. As depicted in **Supplementary Figure 3C**, the expression of OPA1 decreased in the OxyHb group (Vehicle vs OxyHb, F=14.88, P<0.0001), but the tendency was reversed by the administration of PTE (OxyHb+PTE vs OxyHb, F=14.88, P=0.0186). However, PTE failed to up-regulate OPA1 in Nrf2 knockdown cells (OxyHb+PTE+siNrf2 vs OxyHb, F=14.88, P>0.9999), suggesting PTE promote OPA1 expression in a Nrf2 dependent manner. This effect was further substantiated by conducting PCR analysis of the target genes of Nrf2 (HO-1, NQO1, and SOD-1) in the *in vitro* model (HO-1: Vehicle vs OxyHb, F=13.66, P<0.0001, OxyHb+PTE vs OxyHb, F=13.66, P=0.0047, OxyHb+PTE+siNrf2 vs OxyHb, F=13.66, P>0.9999, OxyHb+PTE vs OxyHb+PTE+siNrf2, F=13.66, P=0.0324; NQO1: Vehicle vs OxyHb, F=86.75, P<0.0001, OxyHb+PTE vs OxyHb, F=86.75, P<0.0001, OxyHb+PTE+siNrf2 vs OxyHb, F=86.75, P>0.9999, OxyHb+PTE vs OxyHb+PTE+siNrf2, F=86.75, P<0.0001; SOD-1: Vehicle vs OxyHb, F=28.59, P<0.0001, OxyHb+PTE vs OxyHb, F=28.59, P=0.0085, OxyHb+PTE+siNrf2 vs OxyHb, F=28.59, P>0.9999, OxyHb+PTE vs OxyHb+PTE+siNrf2, F=28.59, P=0.0041). Furthermore, we utilized flow cytometry to isolate microglial cells in the *in vivo* model, and the results revealed a significant reduction in mRNA levels of HO-1, NQO1, and SOD-1 after ICH compared to the Sham group (HO-1: F=17.79, P<0.0001; NQO1: F=85.56, P<0.0001; SOD-1: F=35.51, P<0.0001), while this downward trend was effectively reversed upon PTE administration (HO-1: F=17.79, P=0.024; NQO1: F=85.56, P<0.0001; SOD-1: F=35.51, P=0.0014) (**Supplementary Figure 3G–I**). Taken together, we propose that PTE reversed the downregulation of OPA1 expression after ICH, at least in part, through activating the Nrf2 pathway. Moreover, we found that OPA1 knockdown using siRNA technology enhanced the susceptibility to inflammation in BV2s under OxyHb stimulation (**Supplementary Figure 4**). Detailly, we observed a significant increase in the expression of proinflammatory cytokines, including IL-1β, IL-18, IL-6 and TNF-α, in the OPA1 knockdown group compared to the control group upon OxyHb treatment (IL-6, F=13.07, P=0.0010; IL-1β, F=39.01, P<0.0001; IL-18, F=7.388, P=0.0122; TNF-α, F=8.725, P=0.0072). Based on those results, we speculated that the neuroprotective effect of PTE was mediated by the pathway related to OPA1-dependent mitochondrial fusion, and subsequently verified this in further experiments.

### 3.5 Knockdown of OPA1 countered the protective effects of PTE on mitochondrial morphology and function *in vitro*

**Figure 5:**
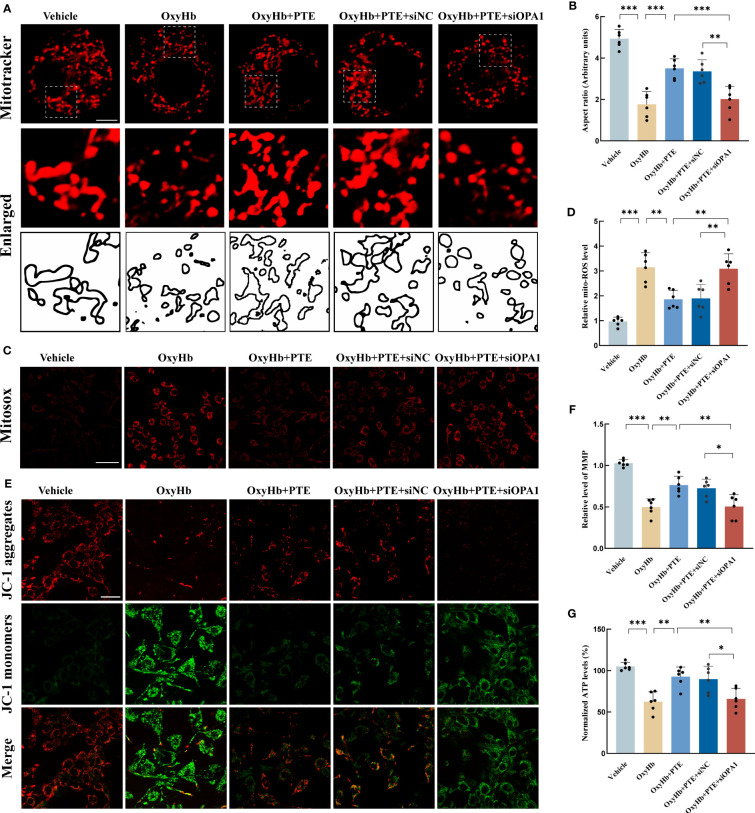
Knockdown of OPA1 abolished the protective effects of PTE on mitochondrial morphology and function *in vitro*. **(A, B)** Mitochondrial morphology analysis in BV2 line of WT and OPA1 knockdown after different treatments, respectively. Mitochondrial morphology was stained by MitoTracker and mitochondrial fragmentation was quantified as aspect ratio in each group. Scale bar, 10 μm. Each micrograph next to the boxed area shows an amplified version of the white square. The OPA1 knockdown BV2 cell line neutralized the PTE-induced increase of mitochondrial aspect ratio. **(C, D)** Measurement of mitochondrial ROS generation by mitoSOX staining. Scale bar, 50 μm. The OPA1 knockdown BV2 cell line counteracted the PTE-induced elevation of ROS in mitochondria. **(E)** Representative confocal microscopy images of JC-1 staining, Scale bar, 20 μm. **(F)** Representative fluorescence staining of JC-1 aggregates (red)/JC-1 monomers (green) illustrating the MMP, with higher red fluorescence intensity indicating higher MMP. **(G)** Consistent with the above results, ATP production also showed similar trends, all indicating that OPA1 knockout countered the PTE-induced protective effect on mitochondrial quality control. Values are expressed as mean ± SD (n = 6 per group). Significance was determined by one-way ANOVA **(B, D, F, G)** with Bonferroni *post hoc* tests. *P < 0.05, **P < 0.01, ***P < 0.001.

To investigate the role of OPA1 in the protective effect of PTE on mitochondrial morphology, we knocked down OPA1 in BV2 microglia using siRNA technology. Then, we explored the changes in mitochondrial morphology in BV2 as described above. As shown in **Figure 5A**, the aspect ratio decreased in the OxyHb group, which was reversed by PTE treatment (**Figures 5A, B**; OxyHb vs OxyHb+PTE, F=32.88, P=0.0001). While in OPA1 knockdown cell lines, PTE couldn’t exert this effect (OxyHb+PTE vs OxyHb+PTE+siOPA1, F=32.88, P=0.0008), suggesting that OPA1 knockdown diminished the protective effect of PTE on mitochondrial morphology *in vitro*. Since mitochondrial dynamics is a necessary process that maintains normal mitochondrial function (20, 51), the effect of PTE on mitochondrial function was also evaluated in BV2. MitoSOX staining showed that after OxyHb treatment, PTE significantly decreased mitochondrial ROS production (**Figures 5C, D**; OxyHb vs OxyHb+PTE, F=21.80, P=0.0010). Then, MMP was measured by JC-1 staining. It was also observed that PTE reversed the downregulation of MMP after OxyHb treatment (**Figures 5E, F**; OxyHb vs OxyHb+PTE, F=25.83, P=0.0020). Moreover, similar results were observed in ATP (**Figure 5G**);, suggesting that PTE also protected against functional impairment of mitochondria efficiently. We further explored those indicators of mitochondrial function in OPA1 knockdown BV2 cells. Likewise, we observed that after OxyHb treatment, PTE-mediated reduction in mitochondrial ROS production and elevation in MMP and ATP level were negated in OPA1 knockdown BV2 cells (ROS: OxyHb+PTE vs OxyHb+PTE+siOPA1, F=21.80, P=0.0017; MMP: OxyHb+PTE vs OxyHb+PTE+siOPA1, F=25.83, P=0.0026; ATP: OxyHb+PTE vs OxyHb+PTE+siOPA1, F=14.26, p=0.0092), indicating that OPA1 knockdown countered the protective effects of PTE on mitochondrial function. These results indicated that PTE alleviated mitochondrial morphological damage and protected mitochondrial function in an OPA1-dependent manner.

### 3.6 Microglial OPA1 knockout countered the protective effects of PTE on neuroinflammation in mice

**Figure 6:**
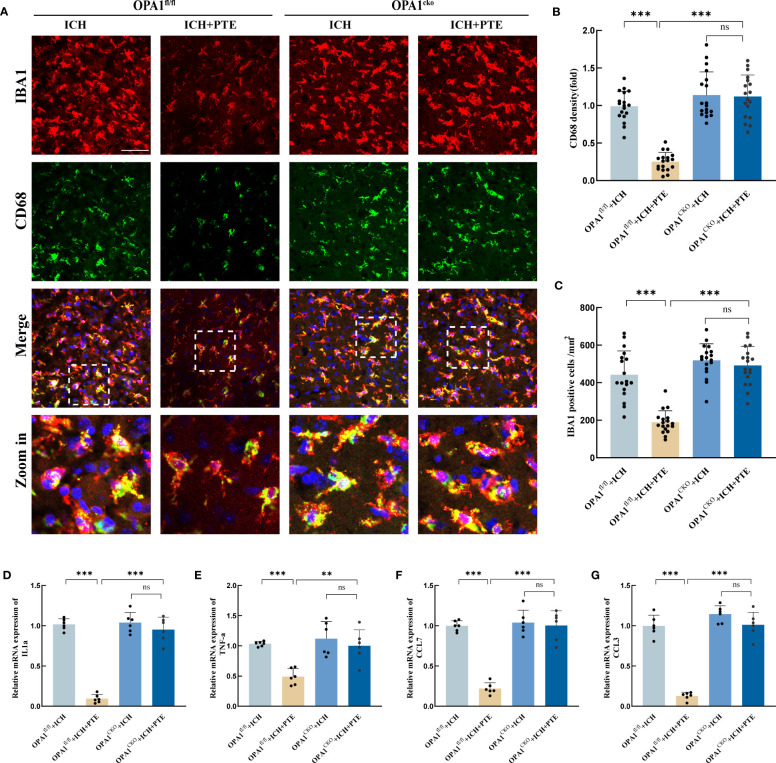
PTE failed to attenuate neuroinflammation after ICH in microglial OPA1 knockout mice. **(A)** Immunofluorescence staining for Iba1(red) with CD68 (green) in OPA1^fl/fl^ and OPA1^CKO^ mice after different treatments, respectively, revealing the level of microglia in each group. Scale bar, 100 μm. **(B)** Quantification analysis of the relative fluorescence intensity in microglia (18 fields of view). **(C)** The number of microglia was counted (18 fields of view). **(D–G)** qPCR analysis of TNF-α, IL-1α, CCL7 and CCL3. Values are expressed as mean ± SD (n =6 per group). Significance was determined by two-way ANOVA **(C–H)** with Bonferroni *post hoc* tests. *P < 0.05, **P < 0.01, ***P < 0.001, ns, no significance.

To further confirm the role of OPA1 in the anti-inflammatory effects of PTE, we generated OPA1^CKO^ mice by crossbreeding OPA1^fl/fl^ mice with CX3CR1-CreERT2 mice. This model expresses Cre recombinase under the control of the CX3CR1 promoter, which is specific to microglia cells in the brain. The tamoxifen-inducible CreERT2 system allows for temporal control of OPA1 deletion. The genotype of the mice was confirmed by the identification map of OPA1^CKO^ mice (**Supplementary Figure 5A**). Additionally, we validated the gene knockout at the mRNA and protein levels after isolating microglia using PCR and Western blot, respectively (**Supplementary Figures 5B, C**). The results revealed a significant reduction in both mRNA and protein expressions of OPA1 in microglia in OPA1^CKO^ mice (mRNA level: t=25.76, P<0.0001; Protein level: t=29.54, P<0.0001). Then we measured microglial proinflammatory activities in both OPA1^CKO^ and OPA1^fl/fl^ mice models. First, we found that after ICH, the inhibitory effect of PTE on microglia-mediated neuroinflammation was abolished in OPA1^CKO^ mice, compared to that in OPA1^fl/fl^ mice (**Figures 6A–C**),. Specifically, PTE significantly suppressed the microglial activity in OPA1^fl/fl^ mice after ICH, as indicated by the decreased relative fluorescence intensity of CD68 and numbers of microglia, whereas PTE did not exert those effects in OPA1^CKO^ mice (CD68 density: OPA1^fl/fl^+ICH vs OPA1^fl/fl^+ICH+PTE, F=43.95, P<0.0001; OPA1^fl/fl^+ICH+PTE vs OPA1^CKO^+ICH+PTE, F=43.95, P<0.0001; Numbers of microglia: OPA1^fl/fl^+ICH vs OPA1^fl/fl^+ICH+PTE, F=37.08, P<0.0001; OPA1^fl/fl^+ICH+PTE vs OPA1^CKO^+ICH+PTE, F=37.08, P<0.0001). Meanwhile, PTE significantly suppressed the expression of IL1α, TNF-α, CCL7 and CCL3 in OPA1^fl/fl^ mice after ICH, but not in OPA1^CKO^ mice (**Figures 6D–G**; IL1α: OPA1^fl/fl^+ICH vs OPA1^fl/fl^+ICH+PTE, F=128.5, P<0.0001; OPA1^fl/fl^+ICH+PTE vs OPA1^CKO^+ICH+PTE, F=128.5, P<0.0001; TNF-α: OPA1^fl/fl^+ICH vs OPA1^fl/fl^+ICH+PTE, F=15.58, P=0.0011; OPA1^fl/fl^+ICH+PTE vs OPA1^CKO^+ICH+PTE, F=15.58, P=0.002; CCL7: OPA1^fl/fl^+ICH vs OPA1^fl/fl^+ICH+PTE, F=59.20, P<0.0001; OPA1^fl/fl^+ICH+PTE vs OPA1^CKO^+ICH+PTE, F=59.20, P<0.0001; CCL3: OPA1^fl/fl^+ICH vs OPA1^fl/fl^+ICH+PTE, F=108.6, P<0.0001; OPA1^fl/fl^+ICH+PTE vs OPA1^CKO^+ICH+PTE, F=108.6, P<0.0001). All these results demonstrated that microglia-specific OPA1 knockout counteracted the inhibitory effect of PTE on neuroinflammation, and PTE exerted an anti-inflammatory effect in an OPA1-dependent manner after ICH.

### 3.7 Microglial OPA1 knockout prevented the positive effects of PTE on brain recovery in experimental mice

**Figure 7:**
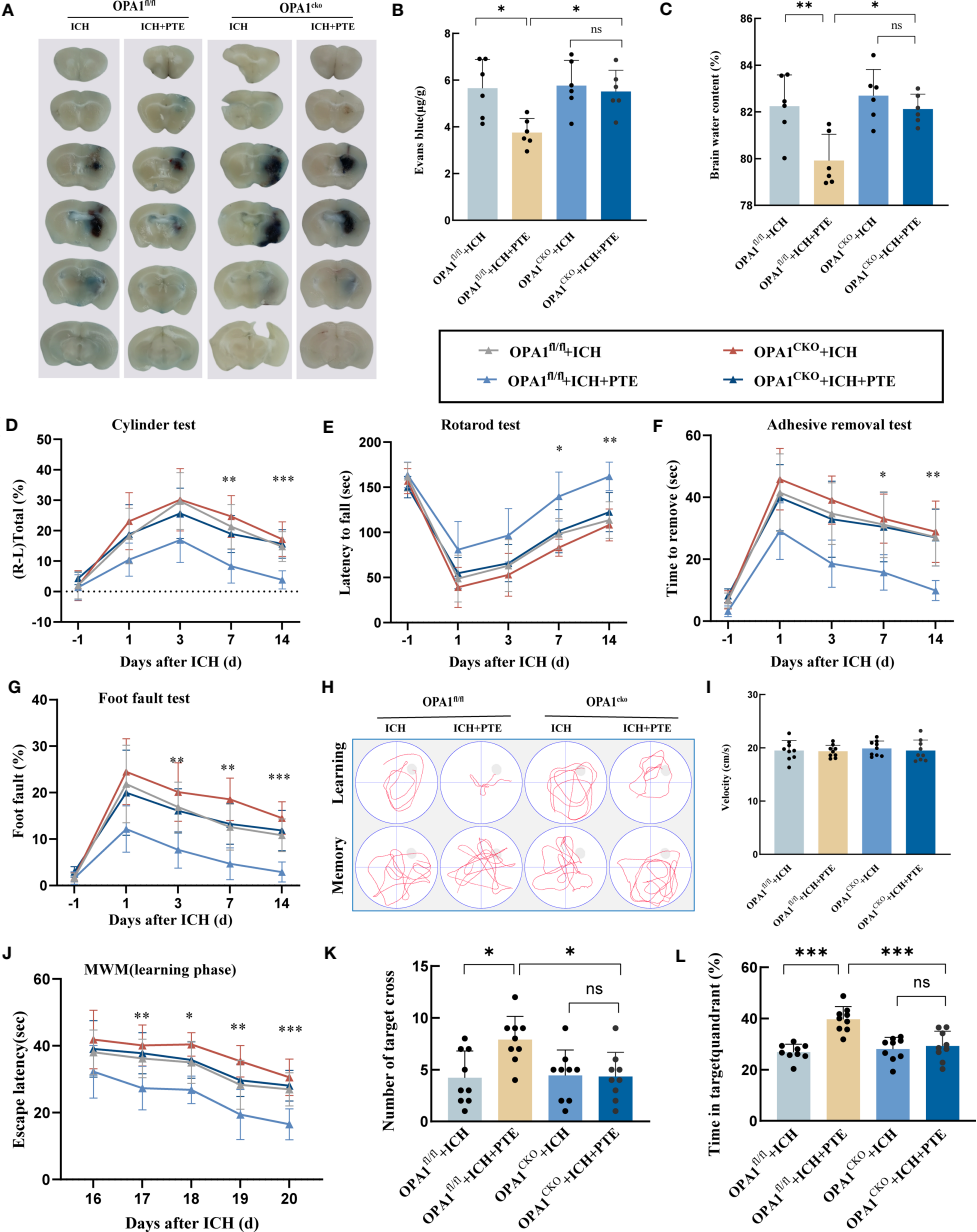
Microglial OPA1 knockout negated the protective effects of PTE on BBB impairment, brain water content, and neurological function scores in mice. **(A, B)** Representative brain sections with Evan blue and its quantification from OPA1^fl/fl^ and OPA1^CKO^ mice after different treatments, respectively (n = 6 per group). **(C)** Brain edema statistical analysis in OPA1^fl/fl^ and OPA1^CKO^ mice after different treatments, respectively (n = 6 per group). **(D–G)** Adhesive removal, cylinder, grid walking and latency to fall test used to assess neurological recovery from OPA1^fl/fl^ and OPA1^CKO^ mice after different treatments, respectively (n = 9 per group). **(H)** Spatial learning and memory were assessed by the Morris water maze. **(I)** Graph showing the swim speed of mice in the different groups during the screening test before the water maze learning sessions. **(J)** Escape latency in the Morris water maze test. **(K, L)** Time spent in the target quadrant and the number of platform crossings during the probe trial in which the target platform is removed (n = 9 per group). All these results indicated that microglial OPA1 knockout countered the protective effects of PTE on neurological recovery after ICH. Values are expressed as mean ± SD. Significance was determined by two-way ANOVA with Bonferroni *post hoc* tests. *P < 0.05, **P < 0.01, ***P < 0.001, ns, no significance. In behavioral experiments, the asterisks on the top indicate single day comparison between the two groups (ICH+PTE group vs ICH group).

**Figure 8:**
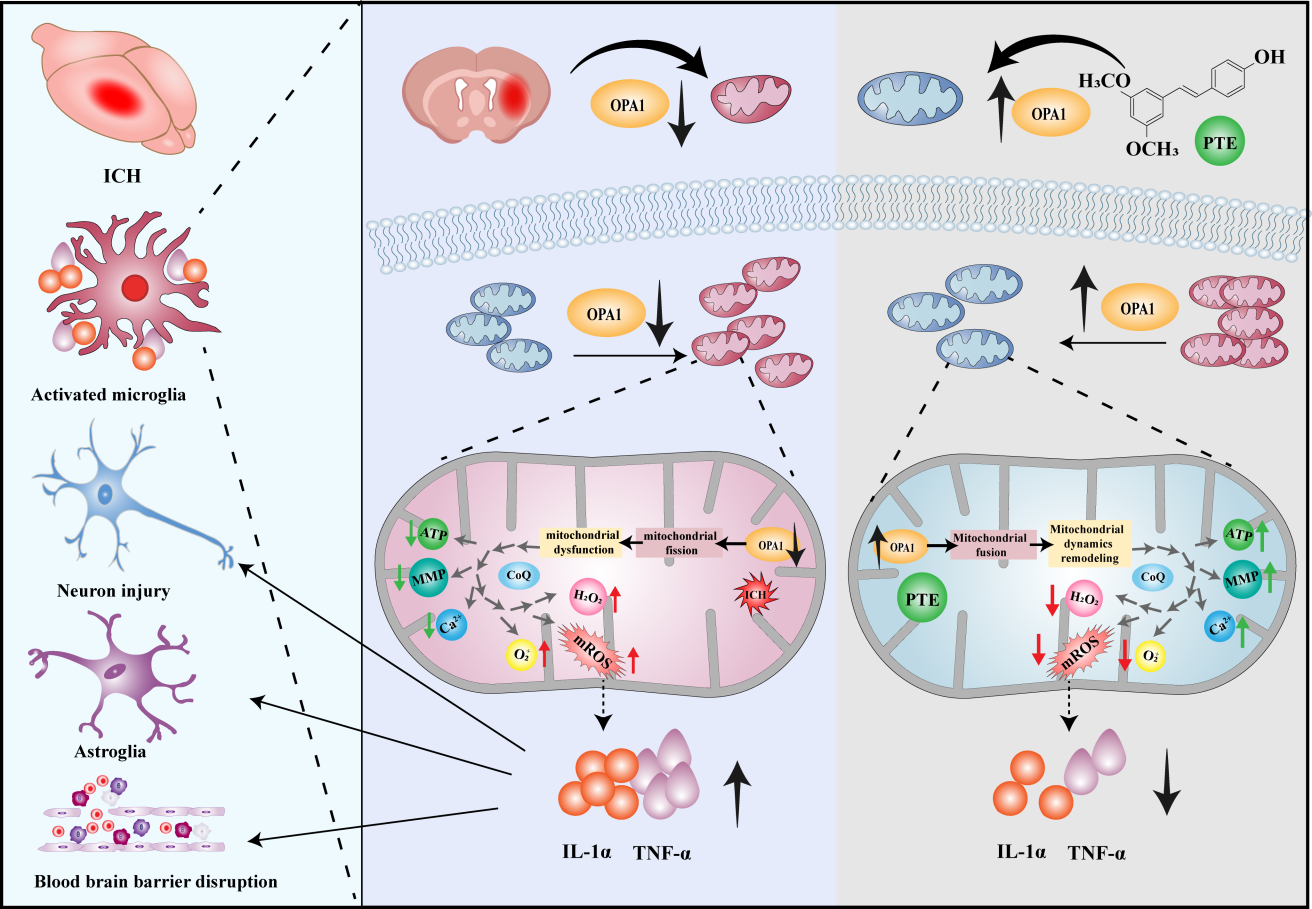
The proposed mechanism of PTE–mediated mitochondrial dynamics remodeling in microglia. PTE could facilitate mitochondrial fusion, remodel mitochondrial dynamics, block excessive superoxide production, and inhibit microglia-mediated neuroinflammation through the upregulation of OPA1.

BBB impairment, brain water content, and neurological outcomes were investigated in both OPA1^CKO^ and OPA1^fl/fl^ mice models. The inhibitory effect of PTE on BBB impairment was observed in OPA1^fl/fl^ mice after ICH, but not in OPA1^CKO^ mice (**Figures 7A, B**; OPA1^fl/fl^+ICH vs. OPA1^fl/fl^+ICH+PTE, F=7.169, P=0.0198; OPA1^fl/fl^+ICH+PTE vs OPA1^CKO^+ICH+PTE, F=7.169, P=0.0354). The same results were found in brain water content that PTE could reduce brain edema after ICH in OPA1^fl/fl^ mice models, but failed to alleviate ICH-induced brain edema in OPA1^CKO^ mice (**Figure 7C**; OPA1^fl/fl^+ICH vs OPA1^fl/fl^+ICH+PTE, F=10.69, P=0.0084; OPA1^fl/fl^+ICH+PTE vs OPA1^CKO^+ICH+PTE, F=10.69, P=0.0134). Then, we conducted neurobehavioral tests to assess the protective effects of PTE on the post-ICH sensorimotor function in OPA1^CKO^ and OPA1^fl/fl^ mice models. As expected, we did not find efficient stimulant effects of PTE on post-ICH recovery of neural function in OPA1^CKO^ mice, as measured in the cylinder, rotarod, adhesive removal and grid walking test (**Figures 7D–G**; Cylinder test: OPA1^fl/fl^+ICH+PTE vs OPA1^CKO^+ICH+PTE, F=13.02, P=0.007; Rotarod test: OPA1^fl/fl^+ICH+PTE vs OPA1^CKO^+ICH+PTE, F=10.03, P=0.004; Adhesive removal test: OPA1^fl/fl^+ICH+PTE vs OPA1^CKO^+ICH+PTE, F=10.05, P=0.0021; Grid walking test: OPA1^fl/fl^+ICH+PTE vs OPA1^CKO^+ICH+PTE, F=14.58, p=0.0006). Furthermore, we conducted the MWM test to assess the long‐term effect of PTE on neurobehaviors. Similarly, the stimulant effects of PTE on post-ICH recovery of learning and memory function were negated in OPA1^CKO^ mice, as shown by escape latency (**Figure 7J**; OPA1^fl/fl^+ICH+PTE vs. OPA1^CKO^+ICH+PTE, F=40.72, P<0.0001), time in the target quadrant and numbers of platform crossing (**Figures 7K, L**; Crossing number: OPA1^fl/fl^+ICH vs OPA1^fl/fl^+ICH+PTE, F=4.876, P=0.0227; Time in target quadrant: OPA1^fl/fl^+ICH vs OPA1^fl/fl^+ICH+PTE, F=20.48, P=0.0002). Together, our data indicated that microglia-specific OPA1 knockout weakened the stimulant effects of PTE on neurological recovery after ICH. At last, a diagram of the molecular mechanism elucidated in this study is shown in **Figure 8**.

## 4 Discussion

In our study, we evaluated the effect of PTE on attenuating inflammatory injury after experimental ICH and investigated its potential mechanism of anti-inflammatory effects after ICH. *In vivo*, PTE played a neuroprotective role by decreasing lesion volume and brain edema, alleviating BBB damage and neuronal apoptosis, and improving neurological outcomes following ICH. It also markedly suppressed ICH-induced neuroinflammation and microglial proinflammatory activities in the peri-hematomal region. *In vitro*, PTE treatment reversed the downregulation of OPA1 in microglia, promoted mitochondrial fusion, restored normal mitochondrial morphology, and reduced mitochondrial fragmentation and superoxide after OxyHb treatment. Moreover, conditionally deleting microglial OPA1 in mice largely countered the effects of PTE on alleviating microglial inflammation, BBB damage, brain edema and neurological impairment following ICH. These results may indicate that PTE is a promising neuroprotective agent for brain injury after ICH, and exerts functions at least in part through OPA1‐related mitochondrial fusion, thereby improving post-ICH outcomes.

The high mortality and disability rates in ICH patients can be attributed to the secondary brain injury caused by hematoma metabolites, primarily characterized by brain edema and inflammation (4, 52). Here, we aim to identify effective therapeutic drugs that can alleviate secondary brain injury and improve neural functional prognosis. PTE, a dimethylated analog of resveratrol, carries broad anti-inflammatory and anti-oxidative stress abilities, which have caught our attention (28). Recent studies have shown promising applications of PTE in CNS diseases (29, 53). Liu et al. reported that PTE acts to suppress neuroinflammation and mitochondrial oxidative stress after SAH and cerebral ischemia-reperfusion injury (27, 29). However, the neuroprotective effects of PTE in ICH remain unknown. In this study, we observed that PTE could markedly promote the recovery of neurological function assessed by the adhesive removal, grid-walking, latency to fall, cylinder and MWM test. These behavioral improvements with PTE treatment were correlated with significantly decreased lesion volume, BBB damage, brain edema and neural apoptosis cortical and hippocampal tissue. Those results provided the first evidence that PTE may be a promising therapeutic candidate for post-ICH brain injury.

The activation of microglia and the resulting neuroinflammatory response are significant factors that contribute to the poor prognosis associated with ICH. Microglial cells reside in the neural parenchyma to safeguard microenvironmental homeostasis (8). Upon ICH onset, microglial cells are triggered by noxious agents or injurious processes and characterized by overgrowth, morphological change, and production of neurotoxic substrates, like TNFα, IL1α, CCL3, etc., causing edema, inflammation and damage of the perihematomal brain tissues (45, 46). To gain a better understanding of the molecular mechanisms regulated by PTE, we utilized RNA sequencing. The results showed that PTE treatment reversed 1021 differentially expressed genes. Additionally, GO enrichment analysis indicated that PTE significantly suppressed inflammation-related processes, highlighting its potential to alleviate inflammatory injury after ICH. Furthermore, PTE reduced the number of reactive microglia, restored microglial hypertrophic morphology, and alleviated microglia-mediated neuroinflammation, suggesting that PTE effectively suppresses microglial activation after ICH injury. These results are consistent with previous findings that highlight PTE’s ability to regulate microglial activation, which is characterized by neurodegeneration and stroke (32, 54). For instance, Wang et al. reported that PTE attenuated LPS-induced microglial activation, thereby reducing the release of proinflammatory factors such as IL-6, IL-1β, and TNF-α in neurodegenerative diseases (54).

Accumulating clinical and preclinical evidence indicates that mitochondria are key players in neuroinflammatory and neurodegenerative diseases, as well as stroke. There is growing evidence for the involvement of both mitochondrial ROS and mitochondrial metabolism in inflammatory microglia activation after ICH. Structurally, balancing mitochondrial dynamics is required to preserve mitochondrial function, which is essential for cells to function normally (51). Gao et al. reported that the persistent microglial activation was accompanied by increased mitochondrial fission and decreased fusion in the ICH mouse model (55). Excessive mitochondrial fission results in mitochondrial fragmentation and triggers cell apoptosis. Another study showed that treatment with Mdivi-1, a mitochondrial fission inhibitor, reduced brain injury and improved neurological function in mice after ICH (56). This treatment also inhibited microglial activation and reduced the levels of proinflammatory cytokines, indicating that targeting mitochondrial fission could alleviate neuroinflammation and improve outcomes after ICH. With regard to the mechanisms, previous studies have shown that PTE is a promising agent for regulating mitochondrial bioenergetic functions and antioxidant capacity (57). However, little attention has been paid to its effects on mitochondrial dynamics and the underlying mechanisms. Our study showed that PTE could reverse ICH-induced collapse of cristae, mitochondrial fragmentation and shrinkage, indicating PTE might inhibit microglial activation and reduces neuroinflammatory response after ICH through remodeling mitochondrial dynamics.

Mitochondrial fission and fusion are two strong determinants of mitochondrial dynamics. Ample evidence demonstrates that GTPases, such as Drp1, Mfn1, Mfn2 and OPA1, can modulate mitochondrial fusion and fission (51). Chaanine et al. demonstrated that Drp1-mediated mitochondrial fission and OPA1-mediated mitochondrial fusion play opposing roles in regulating mitochondrial morphology and function in a mouse model of ischemic stroke (58). In addition, He et al. found that Drp1-dependent mitochondrial fission mediates microglia pyroptosis and neuroinflammation in a mouse model of ICH (59). Jeong et al. demonstrated the pivotal role of Mfn1 and Mfn2 in governing mitochondrial fusion, a process proved to be neuroprotective (60). Our study showed that that PTE significantly enhanced the expression of OPA1 after OxyHb treatment, but had no influence on that of Mfn1, Mfn2, and Drp1. Based on those results, we speculated that the protective effect of PTE on mitochondrial morphology was mediated by the pathway related to OPA1-dependent mitochondrial fusion. To investigate the role of OPA1 in the protective effect of PTE on mitochondria, we knocked down OPA1 using siRNA *in vitro*, and then found that the protective effect of PTE on mitochondrial morphology and functions after OxyHb treatment was abolished. Furthermore, we generated OPA1^CKO^ mice and found that the protective effects of PTE on microglia-mediated neuroinflammation and brain injury were negated after ICH. Together, those results suggest that PTE exerts anti-inflammatory properties through OPA1‐mediated mitochondrial dynamics remodeling.

Several limitations should be noted in our study. Firstly, collagenase, which is widely used to establish the ICH model, induces hemorrhage-like pathological features by degrading the extracellular matrix and disrupting the blood-brain barrier’s integrity. However, ICH manifestations are heterogeneous, with varying hemorrhage locations, durations, and severity levels among patients. Thus, none of the currently prevalent ICH mouse models, including the collagenase-induced ICH and autologous blood-induced ICH models, fully replicate the disease process of ICH injury (3). As a result, we must further refine and validate PTE’s efficiency in conditions that more closely mirror the human condition. Secondly, experimental models are often established in healthy, young rodents that are not exposed to any other medications. However, ICH patients frequently suffer from several severe comorbidities, such as diabetes and cardiovascular diseases, and have taken other drugs before their ICH occurrence. Therefore, we must take these variables into our consideration. Thirdly, we only focused on changes in microglia, but the effects of PTE on microglia-neuron and microglia-astrocyte crosstalk after ICH should be further explored. All these challenges require us to conduct more specific studies with larger sample sizes and more advanced technologies to explore.

## 5 Conclusions

Our study confirms for the first time that PTE is a potential therapeutic agent for ICH and protects against post-ICH secondary brain injury by suppressing microglia-derived inflammation in mice. Meanwhile, we innovatively elucidate the pharmacological mechanism that PTE remodels mitochondrial dynamics, mainly dependent on OPA1‐related mitochondrial fusion.

## Data availability statement

The original contributions presented in the study are included in the article/**Supplementary Material**. Further inquiries can be directed to the corresponding author.

## Ethics statement

The animal study was reviewed and approved by the Institutional Animal Care and Use Committee of the Air Force Medical University.

## Author contributions

GZ and JW conceived and designed the study. YW, QH, XW, HC, JY, YL, JL and QZ acquired and analyzed the data. YW and QH drafted a substantial portion of the manuscript and revised the manuscript. All authors contributed to the article and approved the submitted version.

## Supplementary material

Supplementary Figure 1 Effect of PTE on hippocampal neuronal loss and apoptotic death in the brain following ICH. **(A)** Representative Nissl staining images and quantification illustrating Nissl positively stained neurons in hippocampal CA1, CA2 and CA3 regions. Magnification x30. Scale bar, 20 μm. n=6 mice in each experimental group. Data are represented as mean ± SD and were analyzed by one-way ANOVA with Bonferroni *post hoc* tests. *P<0.05, **P<0.01, ***P<0.001.

Supplementary Figure 2 qPCR was used to validate the trend in the expression of microglial activation marker at the transcriptional level. **(A)** IONS mRNA level. **(B)** CD86 mRNA level. **(C)** CD206 mRNA level. **(D)** Arg1 mRNA level. n=6 mice in each experimental group. Data are represented as mean ± SD and were analyzed by one-way ANOVA with Bonferroni *post hoc* tests. ***P<0.001.

Supplementary Figure 3 PTE enhances Opa1 expression by activating the Nrf2 pathway. **(A)** OPA1 mRNA level in BV2 cells after different treatments. **(B)** The protein expression of Nrf2 in the nuclear and cytosolic fractions. **(C)** OPA1 mRNA level in the BV2 line of WT and Nrf2 knockdown after different treatments, respectively. **(D–F)** The mRNA levels of NQO1, SOD-1, and HO-1 in the *in vitro* model as described above. **(G–I)** We purified microglia from brain tissue using flow cytometry, and detected the expression of the aforementioned target genes of Nrf2. n=6 mice in each experimental group. Data are represented as mean ± SD and were analyzed by one-way ANOVA with Bonferroni *post hoc* tests. *P<0.05, **P<0.01, ***P<0.001.

Supplementary Figure 4 Effect of OPA1 knockdown on susceptibility to inflammation or cytokine production in BV2 microglia under OxyHb stimuli. The concentration of these chemokines (IL-1β, IL-18, IL-6, and TNF-α) was assayed via Elisa. **(A)** Quantitative analysis of IL-6 concentration. **(B)** Quantitative analysis of IL-1β concentration. **(C)** Quantitative analysis of IL-18 concentration. **(D)** Quantitative analysis of TNF-α concentration. n=6 mice in each experimental group. Data are represented as mean ± SD and were analyzed by two-way ANOVA with Bonferroni *post hoc* tests. **P<0.01, ***P<0.001.

Supplementary Figure 5 Identification of the genotype of the mice. **(A)** Agarose gel electrophoresis of the polymerase chain reaction (PCR) products for genotyping of the mouse strains. **(B, C)** Additionally, we validated the gene knockout at the mRNA and protein levels after isolating microglia using PCR and WB, respectively. n=6 mice in each experimental group. Data are represented as mean ± SD and were analyzed by t-tests. ***P<0.001.
