# Supplementary figures and images for: Pterostilbene attenuates microglial inflammation and brain injury after intracerebral hemorrhage in an OPA1-dependent manner
Source: Front Immunol. 2023 Aug 8;14:1172334. doi: 10.3389/fimmu.2023.1172334 (PMC10442819; doi:10.3389/fimmu.2023.1172334)

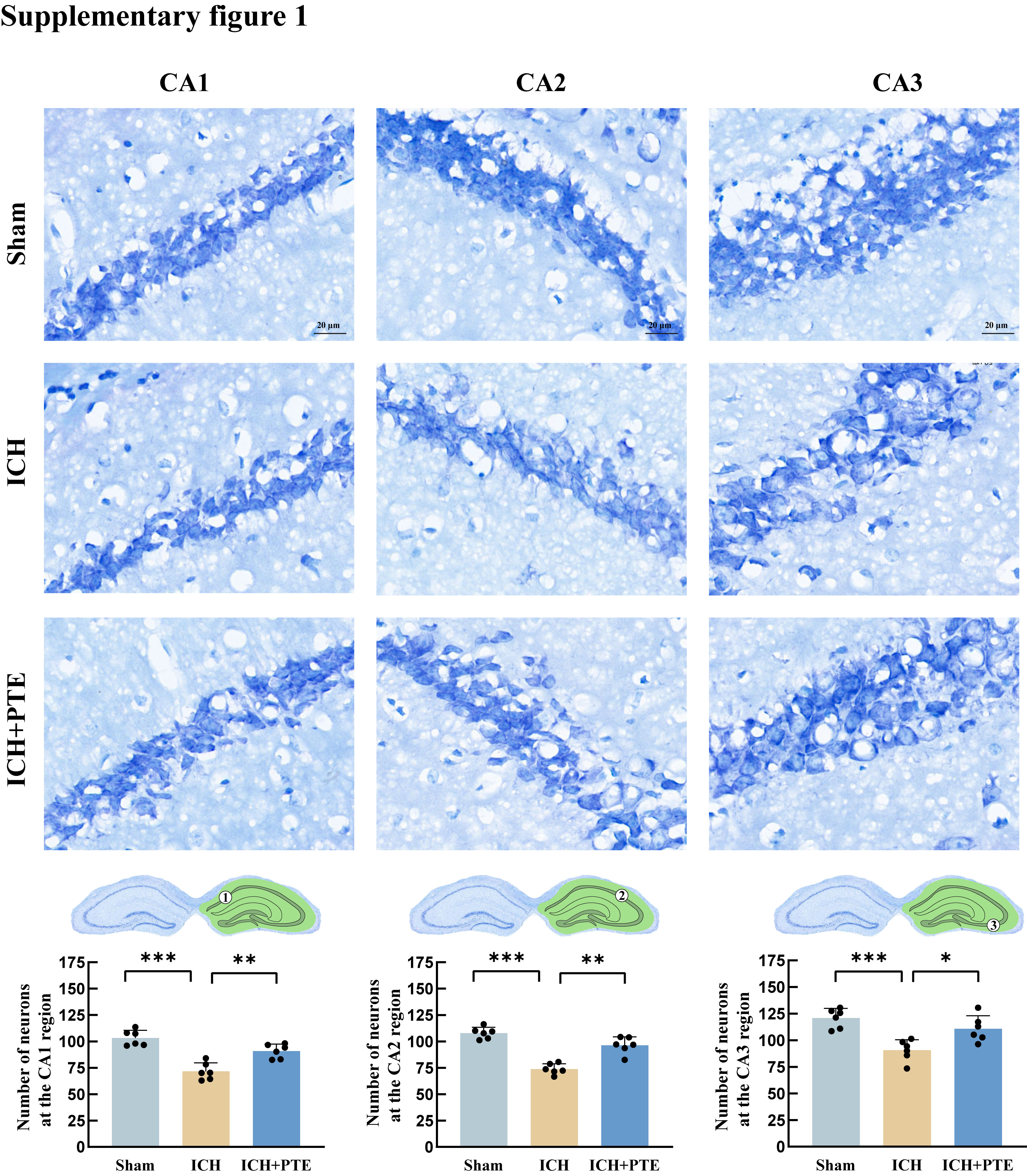

Supplement: Supplementary Figure 1 — Effect of PTE on hippocampal neuronal loss and apoptotic death in the brain following ICH. (A) Representative Nissl staining images and quantification illustrating Nissl positively stained neurons in hippocampal CA1, CA2 and CA3 regions. Magnification x30. Scale bar, 20 μm. n=6 mice in each experimental group. Data are represented as mean ± SD and were analyzed by one-way ANOVA with Bonferroni post hoc tests. *P<0.05, **P<0.01, ***P<0.001. [file Image_1.jpg]

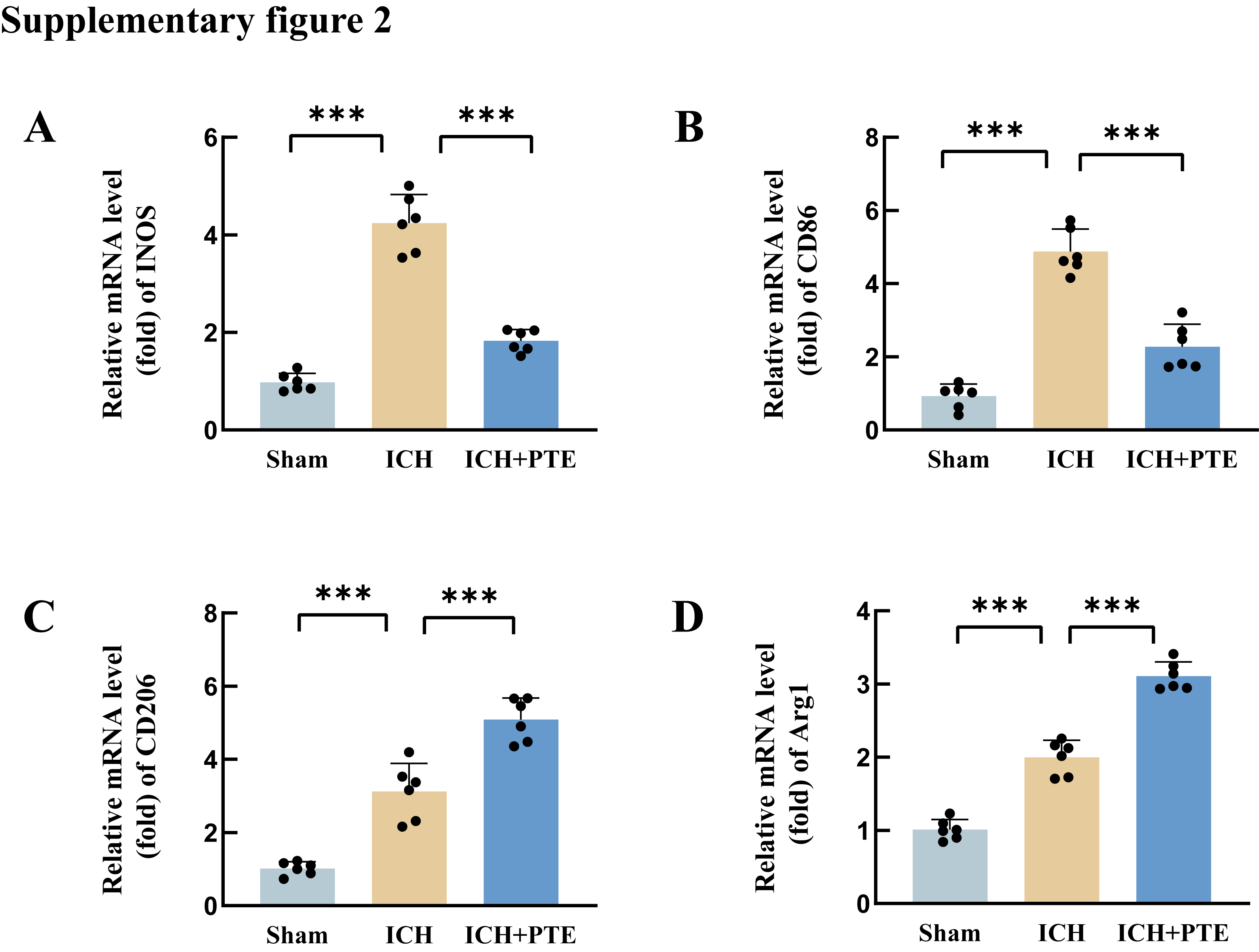

Supplement: Supplementary Figure 2 — qPCR was used to validate the trend in the expression of microglial activation marker at the transcriptional level. (A) IONS mRNA level. (B) CD86 mRNA level. (C) CD206 mRNA level. (D) Arg1 mRNA level. n=6 mice in each experimental group. Data are represented as mean ± SD and were analyzed by one-way ANOVA with Bonferroni post hoc tests. ***P<0.001. [file Image_2.tif]

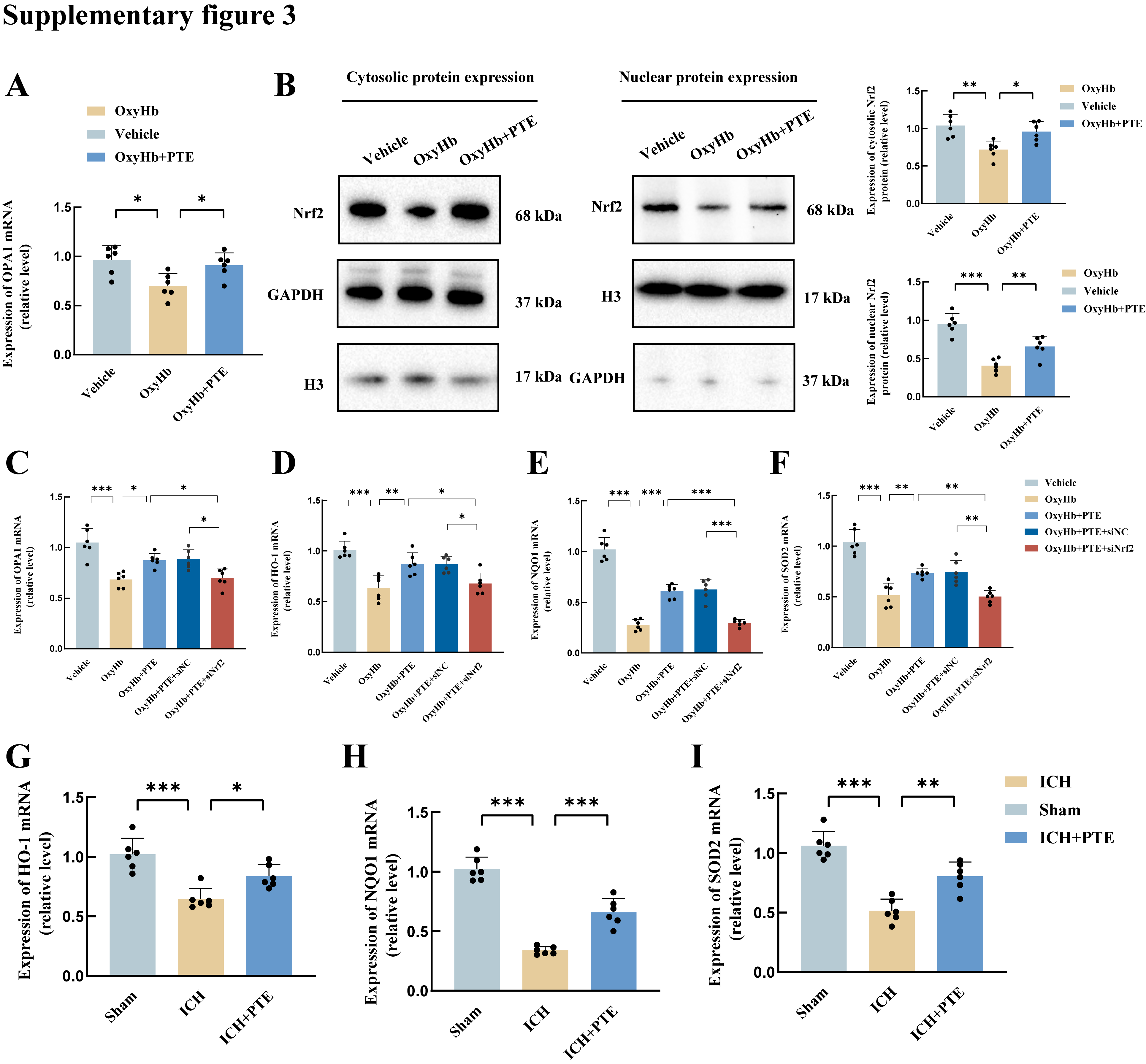

Supplement: Supplementary Figure 3 — PTE enhances Opa1 expression by activating the Nrf2 pathway. (A) OPA1 mRNA level in BV2 cells after different treatments. (B) The protein expression of Nrf2 in the nuclear and cytosolic fractions. (C) OPA1 mRNA level in the BV2 line of WT and Nrf2 knockdown after different treatments, respectively. (D–F) The mRNA levels of NQO1, SOD-1, and HO-1 in the in vitro model as described above. (G–I) We purified microglia from brain tissue using flow cytometry, and detected the expression of the aforementioned target genes of Nrf2. n=6 mice in each experimental group. Data are represented as mean ± SD and were analyzed by one-way ANOVA with Bonferroni post hoc tests. *P<0.05, **P<0.01, ***P<0.001. [file Image_3.tif]

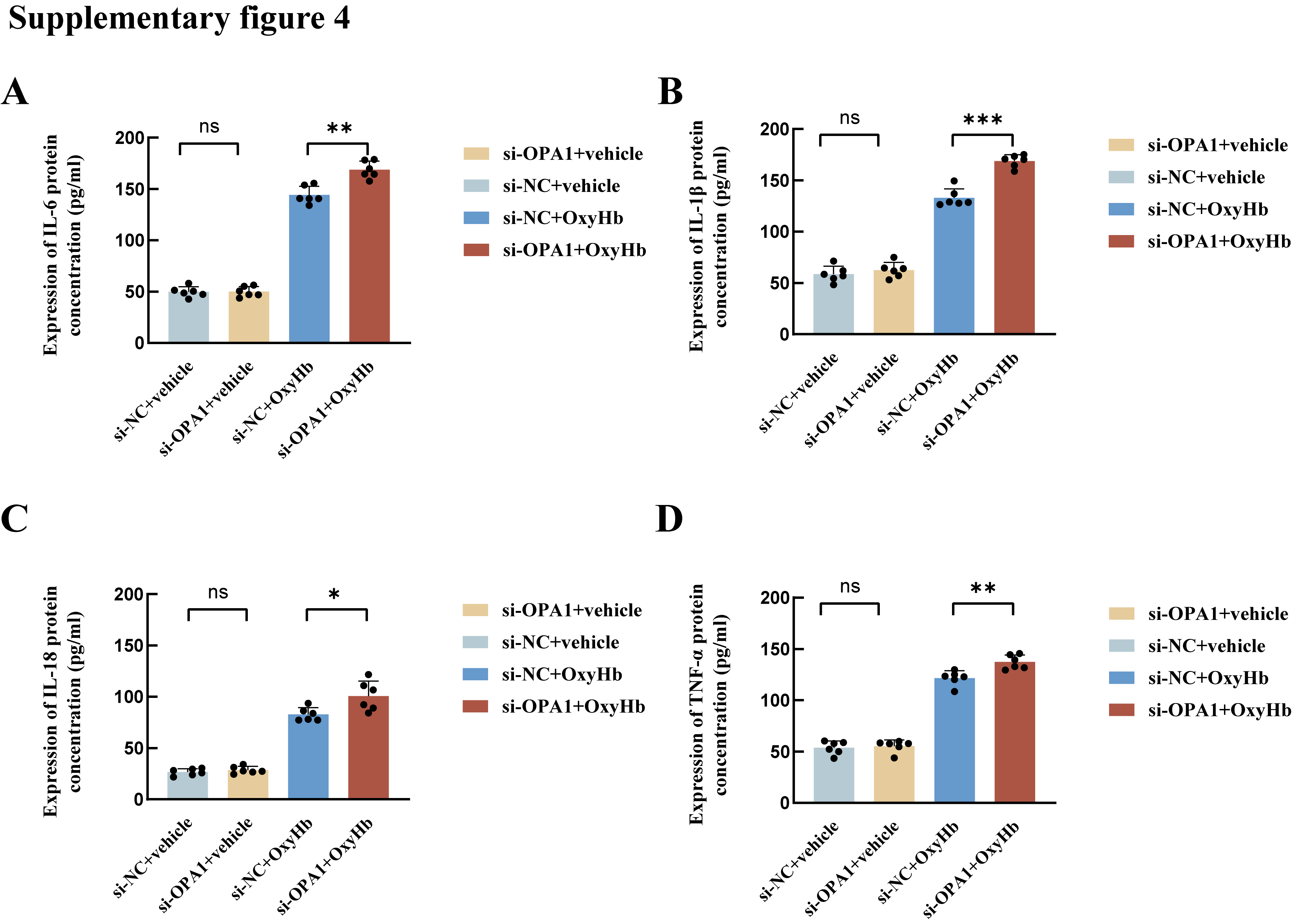

Supplement: Supplementary Figure 4 — Effect of OPA1 knockdown on susceptibility to inflammation or cytokine production in BV2 microglia under OxyHb stimuli. The concentration of these chemokines (IL-1β, IL-18, IL-6, and TNF-α) was assayed via Elisa. (A) Quantitative analysis of IL-6 concentration. (B) Quantitative analysis of IL-1β concentration. (C) Quantitative analysis of IL-18 concentration. (D) Quantitative analysis of TNF-α concentration. n=6 mice in each experimental group. Data are represented as mean ± SD and were analyzed by two-way ANOVA with Bonferroni post hoc tests. **P<0.01, ***P<0.001. [file Image_4.tif]

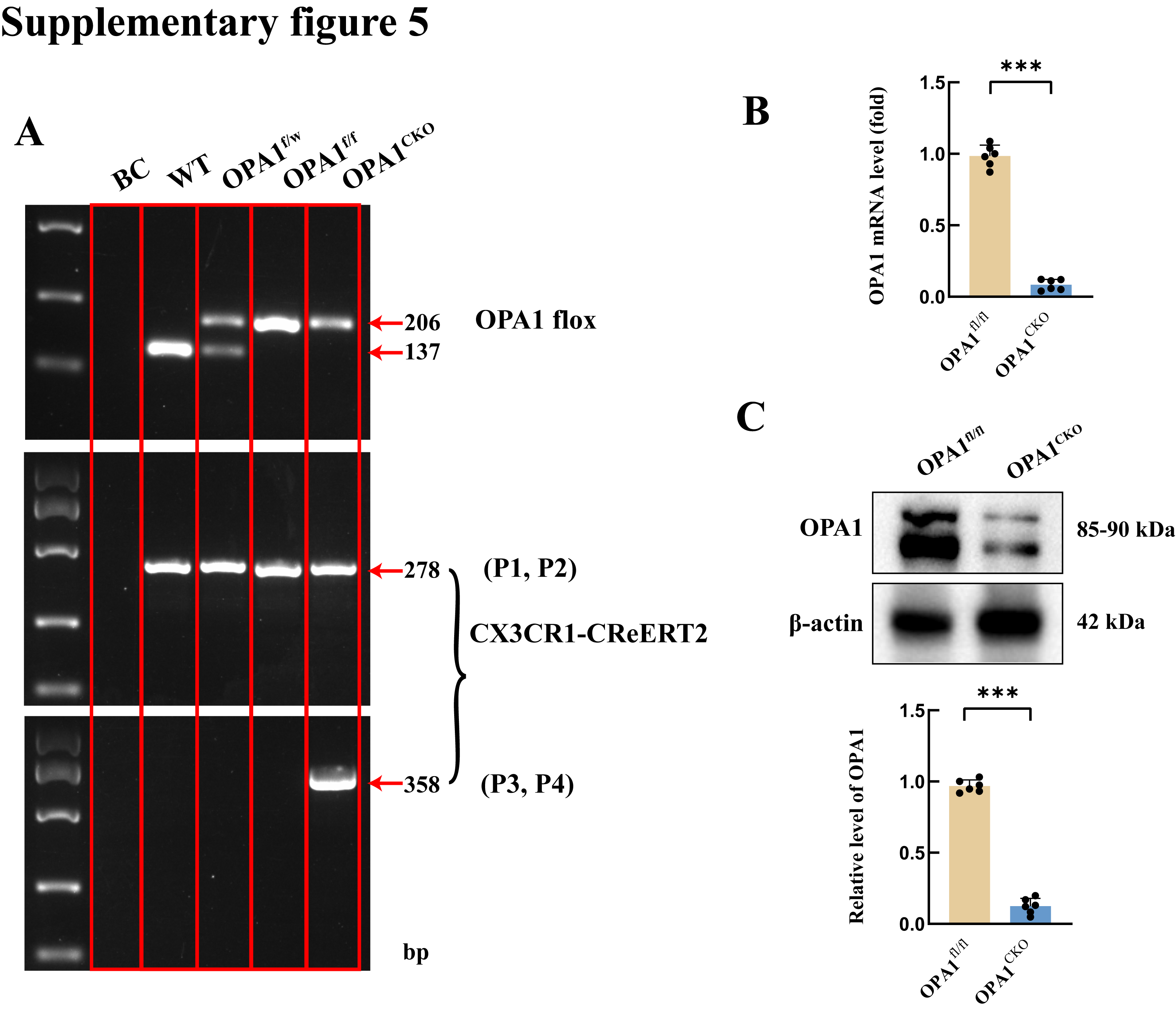

Supplement: Supplementary Figure 5 — Identification of the genotype of the mice. (A) Agarose gel electrophoresis of the polymerase chain reaction (PCR) products for genotyping of the mouse strains. (B, C) Additionally, we validated the gene knockout at the mRNA and protein levels after isolating microglia using PCR and WB, respectively. n=6 mice in each experimental group. Data are represented as mean ± SD and were analyzed by t-tests. ***P<0.001. [file Image_5.tif]
