# Supplementary material for: Pterostilbene attenuates microglial inflammation and brain injury after intracerebral hemorrhage in an OPA1-dependent manner
Source: Front Immunol. 2023 Aug 8;14:1172334. doi: 10.3389/fimmu.2023.1172334 (PMC10442819; doi:10.3389/fimmu.2023.1172334)
Supplement: Supplementary file 6 [file Table_1.docx]

**Table. S1** Sequences of primers and siRNAs used in this study.

| **Gene** | **Primer sequence 5`-3`** |
| --- | --- |
|  | **Forward Reverse** |
| IL-1α TCTCAGATTCACAACTGTTCGTG AGAAAATGAGGTCGGTCTCACTA  TNF-α CAGGCGGTGCCTATGTCTC CGATCACCCCGAAGTTCAGTAG  CCL3 TGTACCATGACACTCTGCAAC CAACGATGAATTGGCGTGGAA  CCL7 CCACATGCTGCTATGTCAAGA ACACCGACTACTGGTGATCCT  OPA1 TGACAAACTTAAGGAGGCTGTG CATTGTGCTGAATAACCCTCAA  CD86 ACGTATTGGAAGGAGATTACAGCT TCTGTCAGCGTTACTATCCCGC  CD206 CTCTGTTCAGCTATTGGACGC CGGAATTTCTGGGATTCAGCTTC  INOS ATGGACCAGTATAAGGCAAGC GCTCTGGATGAGCCTATATTG  Arg1 CTCCAAGCCAAAGTCCTTAGAG AGGAGCTGTCATTAGGGACATC  HO-1 CGACAGCATGTCCCAGGATT TCGCTCTATCTCCTCTTCCAGG  NQO1 CATTCTGAAAGGCTGGTTTGA CTAGCTTTGATCTGGTTGTCG  SOD2 AGGAGAGTTGCTGGAGGCTA AGCGGAATAAGGCCTGTTGTT  β-actin GTGCTATGTTGCTCTAGACTTCG ATGCCACAGGATTCCATACC  si-NC 5’UUCUCCGAACGUGUCACGU dTdT3’  si-OPA1 5’GCUUACAUGCAGAAUCCUA dTdT3’ | |
|  | |
